# Lightweight active back exosuit reduces muscular effort during an hour-long order picking task

**DOI:** 10.1038/s44172-024-00180-w

**Published:** 2024-02-23

**Authors:** Jinwon Chung, D. Adam Quirk, Megan Applegate, Michael Rouleau, Nathalie Degenhardt, Ignacio Galiana, Diane Dalton, Louis N. Awad, Conor J. Walsh

**Affiliations:** 1 John A. Paulson School of Engineering and Applied Sciences, Harvard University, Boston, MA USA; 2 Wyss Institute for Biologically Inspired Engineering, Harvard University, Boston, MA USA; 3 Sargent College of Health & Rehabilitation Science, Boston University, Boston, MA USA

**Keywords:** Mechanical engineering, Biomedical engineering

## Abstract

Occupational back exoskeletons and exosuits aim to reduce low back injuries in the workplace. For these technologies to be adopted, it is important that they provide biomechanical benefits to the wearer and do not disrupt job performance. To address this challenge, here we developed a lightweight, soft, active back exosuit that can autonomously control virtual impedance to apply differing assistance during lowering and lifting. In usability tests, participants rated the exosuit as easy to learn and use and reported feeling confident while wearing it. In an experiment involving an hour-long order picking task we demonstrated that the exosuit reduced peak and median muscle activations in the back by 18% and 20%, respectively. Despite the complexity of the movements required, such as walking, bending, and navigating around obstacles while lifting boxes from under a rack, our controller demonstrated impressive robustness with only 14 mistriggers out of 9600 lifts (0.1%). The results of this research suggest that active exosuit technology has the potential to be a highly usable solution to aid warehouse workers in real-world settings.

## Abstract (web summary)

Chung, Quirk and colleagues present a lightweight active back exosuit to assist workers when lifting. Testing in a simulated one-hour order picking task demonstrates the device has practical usability and reduces back muscle effort by 18%.

## Introduction

According to the National Institute of Occupational Safety and Health (NIOSH), back injuries are the most common workplace injury, representing a large economic burden (100 billion dollars per year) and personal burden in the US^1,2^. Back exos, including exoskeletons and exosuits, offer the potential to mitigate the risk of back injuries, and studies have shown that they can reduce back exertion during squat, stoop, and asymmetrical lifting tasks^3,4^. While their translation to the real world is happening, there is a call to evaluate these devices over longer durations in the field or during work simulation tasks to understand whether these devices provide less benefit or face other usability challenges in diverse workplaces^5,6^.

General factors that challenge exo usability are well established, including device weight, device complexity, joint misalignment, discomfort, restriction, and disruption of movement^3,5,7,8^. The literature highlights certain characteristics that can maximize the benefits of back exos. For a specific device, providing a higher level of assistance can yield greater reductions in back muscle activity^9^. However, added assistance can also present perceptual and biomechanical burdens^9,10^. These burdens can be exacerbated when a back exo must adapt to a diverse array of tasks presented in the workplace^7,8^, as discomfort and movement restriction can arise if assistive forces mismatch task demands^9,11–13^.

To some extent, the relative benefits and burdens of a back exo can be linked to force transmission mechanisms (i.e., rigid vs soft) and actuation strategies (i.e., passive vs active)^7^. Exoskeletons use rigid mechanisms (links and joints) to transfer relatively high assistance to a user by directly applying moments through linkages but have the potential for joint misalignment^7,8^. Mimicking biological muscles, exosuits can apply tensile forces across joints and avoid joint misalignment issues, however, they typically apply lower joint moments than rigid devices^5,7,14^. While some soft devices are perceived to produce less work interruption^15^, exosuit-type designs deliver external forces roughly parallel to the spine to reduce loading in biological muscles^16^, these systems require special attention when anchoring them to the body to reduce unwanted movement and ensure comfort^17^. Passive systems, which use elastic components^5,7^, can be tuned to maximize assistance for specific tasks but impose limited adaptability to diverse tasks^8,13,18^. For these passive systems, a spring needs to be carefully chosen to balance the assistance and the risk of movement restriction or discomfort, in particular for reaching down and walking^9,11,12,19^. On the other hand, active systems, while having the potential of enhanced adaptability through external power sources, tend to exhibit higher weight and complexity, which can negatively influence their usability.

Acknowledging that the utility of back exos is often task-dependent^5,12,13,20,21^, a number of groups have developed creative methods to adapt specific designs to a wider array of well-defined tasks. For example, passive systems that incorporate additional mechanisms such as a clutch or moment arm adjusters to improve adaptability during walking, reaching down, and lifting tasks^5,18,19,22^. On the other hand, active systems have demonstrated improved versatility through adaptive controllers^5,13,18^. For example, a number of controllers, which distinguish between lifting and lowering using motion-based state machines or an on/off switch, can selectively provide more assistance during lifting^23–25^. Other controllers have used loading information from EMG sensors or pressure gloves to scale assistance^26,27^ or predict the kinematic trajectories of lifting motions and adapt the assistive force accordingly^28^.

In a dynamic workplace, an active approach offers the potential for a system that can automatically adapt assistance for a specific user or task. However, the application of adaptive forces could be detrimental if the device mistriggers, applying erroneous forces to the actual task demands, leading to device inefficiencies^13,29^, disruptions to the user’s movement^5^, or the user feeling out of control^30^. For example, order picking tasks, which are common in warehouse operations, involve a series of tasks such as walking, lifting, lowering, and transferring items to build pallets with consumer orders. Warehouse workers often need to carefully position objects to build a pallet or pick and place items under or within shelves or racks, which forces them to walk while bending or avoid obstacles while lifting. The highly varied and dynamic movements associated with these tasks often involve multiple small back movements, posing the challenge of always ensuring correct assistance delivery to the wearer.

As the importance of usability, adaptability, and robustness of back exos is acknowledged for real-world translation, multiple review papers emphasize the need to test back exos under conditions that mimic the intended use environment over an extended period of time^3,21^. An order picking task could be a good use case for back exos since it places high loads on the spine, and warehousing has the highest incidence rates of non-fatal injuries, according to the Bureau of Labor and Statistics^31,32^. To date, only one study provides evidence that a passive back exoskeleton can reduce mean back extensor muscle activity by 10.5% during an order picking task^33^. Another study using the same device found that the device became increasingly uncomfortable over time despite perceived assistance during warehousing work^6^, highlighting the importance of reducing device discomfort as a design goal^5,6,22^. Recently, our lab has demonstrated making a back exosuit more adaptable, delivering assistance based on an individual’s desired direction of movement, can reduce measures of peak back extensor muscle activity (up to 15%) as effectively as a high stiffness elastic during lifting tasks, while mitigating the perception of restriction and discomfort during a deep flexion task^10^. However, these benefits were reported for short-term constrained lifting tasks. In order to establish the viability of this active approach for an order picking task, its robust performance in dynamic environments for a longer period of time must be demonstrated.

In this paper, we present a back exosuit to assist common warehouse operations. The back exosuit is lightweight and flexible, using an integrated ribbon-driven system to apply active assistance across the back and the hips. An adaptive impedance controller modulates assistance based on the magnitude and direction of user movement. A transition phase within the controller provides a smooth transition between flexion and extension assistance and reduces the likelihood of mistriggers under complex and dynamic environments. The usability of the back exosuit was evaluated by administering standard usability surveys after subjects completed a series of everyday tasks while wearing the exosuit, as well as timed donning and doffing. The biomechanical efficacy and the robustness of the exosuit controller was tested during an hour-long order picking protocol that emulated warehouse operations to provide a proof in principle towards the potential of this device for real-world use.

## Results

### Soft exosuit with high usability: lightweight, active, adaptive, and robust

**Fig. 1:**
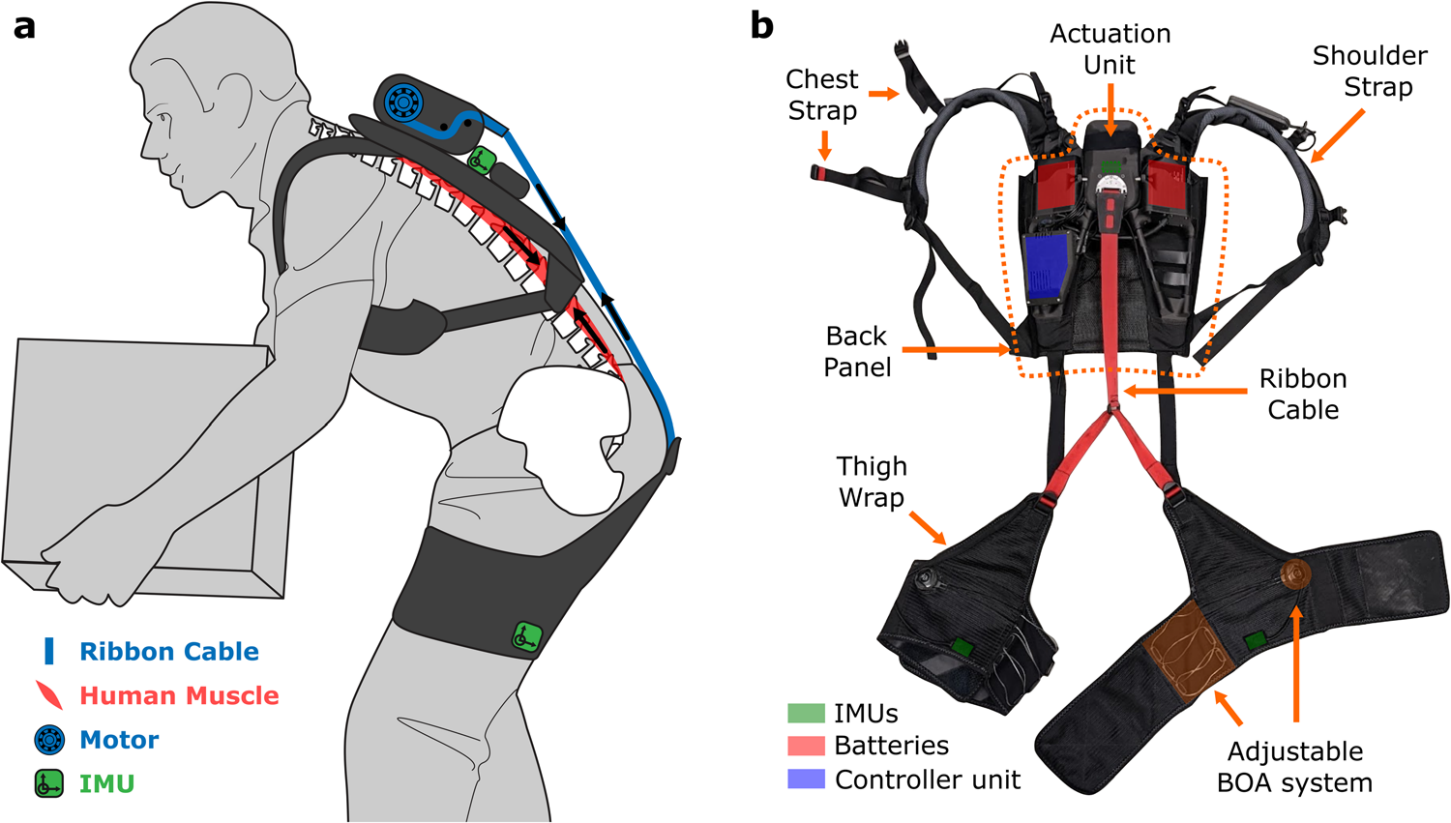
Back exosuit. **a** The back exosuit acts as an external muscle by applying assistive force to the user using a motor and a ribbon cable (blue), via an Inertial Measurement Unit (IMU)-informed controller (green) that acts in parallel with human erector spinae muscles (red). **b** The exosuit is composed of the back panel assembly and the thigh wraps containing important components (green. red, or blue shading). The back panel assembly includes the actuation unit, the controller unit, the shoulder straps, and the chest straps. Three IMU sensors are housed within the actuation unit, and the thigh wraps. The shoulder straps, the chest straps, and the adjustable BOA system in the thigh wraps (orange shading) enable it to accommodate a wide range of body sizes.

In its design, our soft active exosuit aimed to reduce potential joint misalignment and autonomously provide adaptive assistance via lightweight active actuation. This was achieved through a soft textile-based design with an integrated lightweight actuator that modulates the tension of an external ribbon cable spanning the back and hips (Fig. 1a). Three inertial measurement units (IMUs), one on the back and one on each thigh, measure the kinematics of the wearer. The tension on the ribbon cable is measured by a load cell, enabling closed-loop adaptive impedance control. The actuation module, controller unit, batteries, and sensors are integrated into the functional apparel, making the exosuit easy to wear and use (Fig. 1b). The high torque density motor with a lightweight torque amplification pulley system ensures the exosuit is lightweight (2.7 kg) with high overall torque density (11.1 Nm kg^−1^) among other untethered active devices published in the field (Table S1). By utilizing the functional apparel for force transmission, the exosuit did not significantly restrict lateral plane range of motion (RoM) and minimally restricted transverse plane RoM by 4.3° (4.5%) (T(9) = 3.14 *p* = 0.012) compared to movement without the suit (Table S2) (See supplementary text for details).

**Fig. 2:**
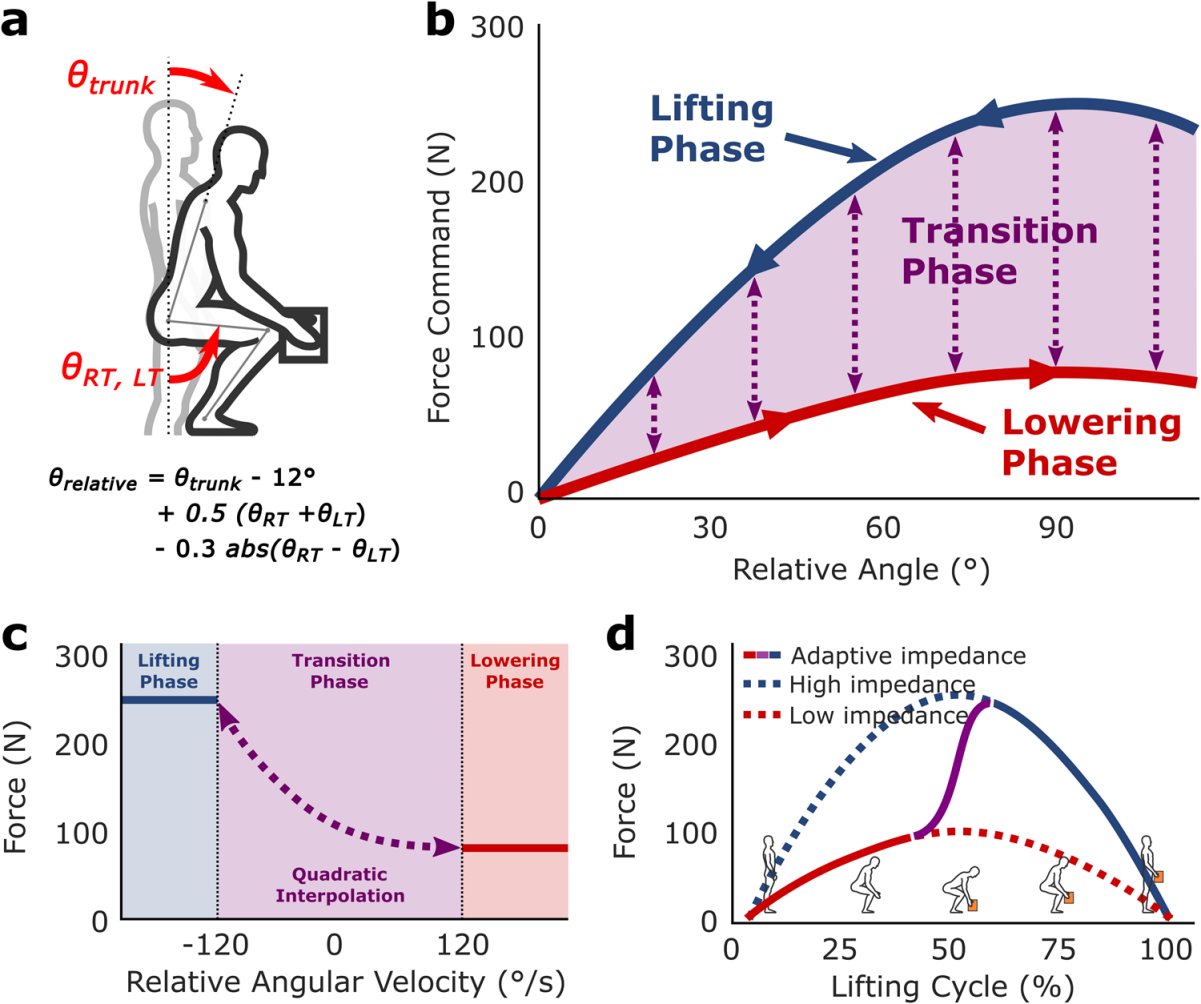
Adaptive impedance controller. **a** The exosuit controller is informed by IMU-measured relative angle between the trunk and the thighs. **b** Assistive forces generated by the exosuit were commanded with an impedance function dependent on the relative angle and phase of motion determining when a participant is lowering (red), lifting (blue), and is in a state of transition between these phases (purple). **c** The movement phase was classified using angular velocity thresholds. At a given angle, lifting and lowering assistance are calculated first, then the transition assistance is quadratically interpolated between them. A wide interpolated transition phase (purple block) of 120 ° s-1 was chosen to prevent mistriggering. **d** Considering the adaptive impedance modulation strategy in the time domain (solid line), our controller was designed to make a smooth transition between a low impedance state when lowering and a high impedance state when lifting. All figures above are generated using idealized data to illustrate the adaptive impedance controller. Subplots c and d are developed with the trunk transitioning from flexion (lowering) to extension (lifting) at approximately 90°. The measured force data is in Fig. 3.

We developed the adaptive impedance controller to assist with lifting without restricting movement or lowering in two ways. First, the controller modulates the virtual impedance of the exosuit based on relative trunk angle and angular velocity (Figs. 2a and S1). Specifically, the exosuit applies higher impedance as a function of relative trunk angle during the lifting phase and lower impedance during the lowering phase, where the phases are determined by relative angular velocity (Fig. 2b, c). Second, to accommodate for the change in trunk moment arm length while bending^26^, we delivered non-linear assistive forces based on a sine curve, such that the exosuit would yield peak assistance (250 N or 30 Nm) once the trunk exceeds a relative angle of 90° (Fig. 2b). Through this approach, our system delivered asymmetric assistive forces as a user is lifting and lowering (Fig. 2d).

To reduce the likelihood of mistriggers, the controller incorporates a transition phase between the lowering and the lifting phases. This transition phase employs a quadratic function of the relative angular velocity to interpolate the force command, gradually transitioning between the assistance levels for lowering and lifting (Fig. 2b, c). Fig. S1 illustrates how the transition forces are determined at 90° and 40° angles as examples. Initially, the lifting phase force (F_lift_) and lowering phase force (F_lower_) are computed based on the relative angle (Fig. 2b). Subsequently, the transition phase force is calculated as a quadratic function of relative angular velocity. The three coefficients of the quadratic function Q, corresponding to a specific relative angle, are carefully chosen to satisfy three constraints: Q(−120° s-1) = F_lift_, Q(120° s-1) = F_lower_, and Q’(120° s-1) = 0 (Fig. 2c).

**Fig. 3:**
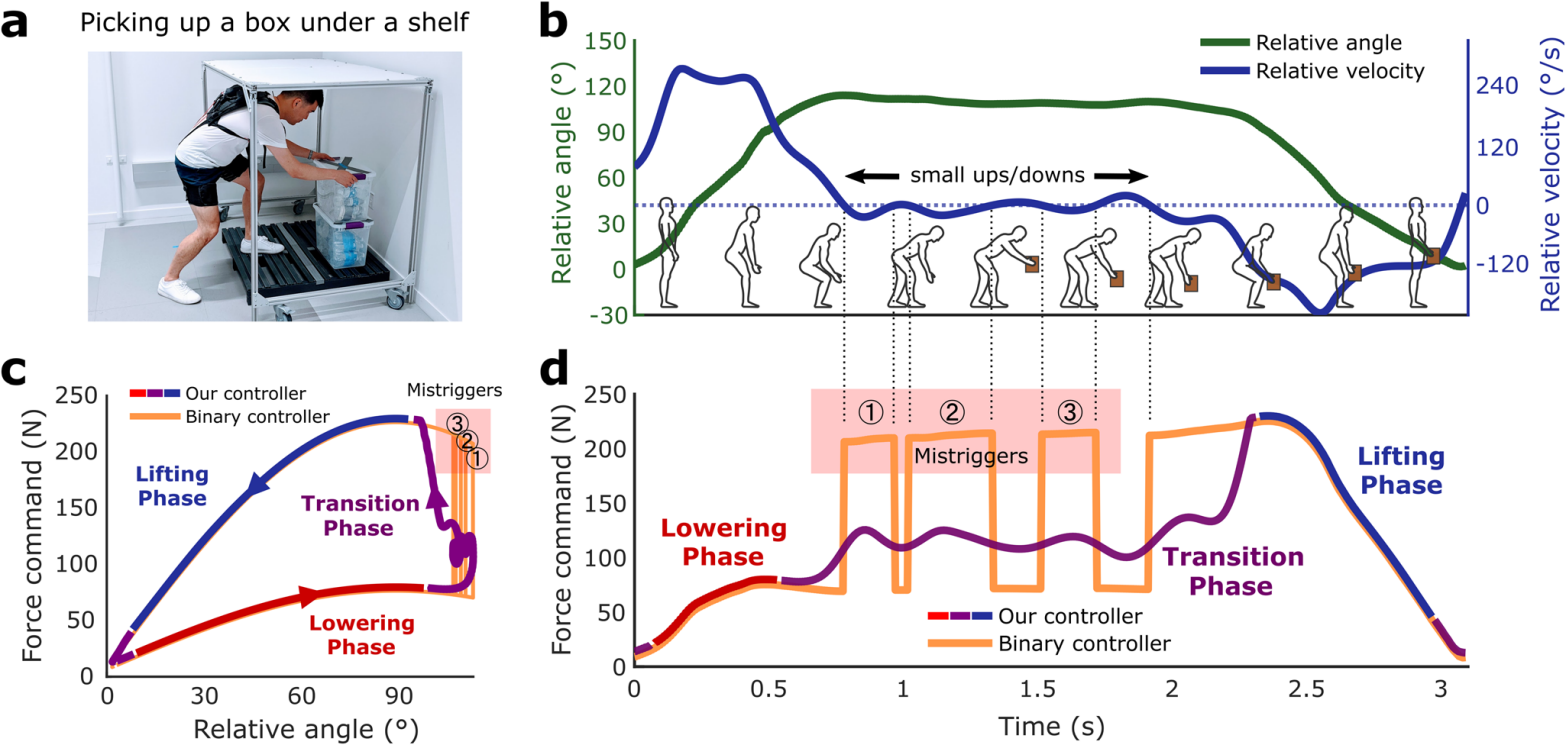
Picking up a box under a shelf. **a** Trunk motion becomes more complex when a participant picks an object under a shelf. **b** These tasks have complex trunk angular kinematics (green line), and angular velocities (blue line) in the time domain that reflect object repositioning (black arrow segment). **c** Depending on the controller, while periods of lifting (blue line) and lowering (red line) can be well-defined, repositioning events can result in the abrupt application of sudden force if a controller mistriggers similar to a binary controller without a transition phase (orange line) in the impedance domain that are avoided with controllers with larger uncertainty (purple line). **d** A binary controller (orange) could have multiple mistriggers in the time domain, which does not occur in our controller with a wide transition window (purple). With the exception of the binary controller data, all the data points presented in the figures are obtained through direct measurement during the protocol. In subplot c and d, the binary controller force profile is represented by simulated data points derived from representative kinematic data.

A wide window for interpolation (±120° s-1) helps to ensure that the force profile changes from low to high assistance once a user was certain of their desired movement direction, whereas a force profile generated by a binary controller without the transition phase would be more likely to result in mistriggers (details of the binary controller in the supplementary material), especially during ambiguous movements (i.e. small changes in direction) such as picking up an item under a shelf (Fig. 3a, b). We define a mistrigger if the controller switches between lowering and lifting assistance more than once within a single lift as the binary controller (orange line) does in Fig. 3c, d.

### Usability test: easy to put on and off, intuitive to use

We evaluated the usability of the back exosuit with ten participants, including six novice users, who had never worn the back exosuit before, and four experts. During the usability test (Movie S1), participants donned the exosuit independently, checked their range of motion, walked around, transferred boxes, and took it off (See methods for details). They completed a system usability scale (SUS) at the end of the experiment^34^. On a scale of 1–5, participants reported that the exosuit was not complex (1.2 ± 0.4) and easy to learn (4.9 ± 0.3), and users felt confident (4.8 ± 0.4) while using it (Tables 1 and S3). Overall, the exosuit achieved an excellent system usability score (SUS) of 92.8 ± 5.3 (Table 1). On average, participants took 35.6 ± 2.5 s to independently wear the exosuit and an additional 10 s for performing the first lift after donning (Table 1). Doffing took approximately 7 seconds (Tables 1 and S3). Qualitatively, novice and expert participants were similar for most metrics (Table 1), but novice users took 6.5 seconds longer to put on the device than experts.

**Table 1:** Overall system usability test results

Usability (1–5: Strongly disagree—strongly agree)	All (*N* = 10)	Range (*N* = 10)	Novice (*N* = 6)	Expert (*N* = 4)
I think the exosuit is complex to use	1.20 ± 0.42	1–2	1.16 ± 0.41	1.25 ± 0.50
I think most users can quickly learn to use the exosuit	4.90 ± 0.32	4–5	4.83 ± 0.41	5.00 ± 0.00
I am confident when using the exosuit	4.80 ± 0.42	4–5	4.67 ± 0.52	5.00 ± 0.00
Overall System Usability Score (SUS)	92.8 ± 5.3	82.5–100	92.9 ± 4.3	92.5 ± 7.4

Test category	A- ll (*N* = 10)	Range (*N* = 10)	Novice (*N* = 6)	Expert (*N* = 4)
Time-To-Don (s)	35.6 ± 2.5	24.7–49.6	38.2 ± 3.2	31.7 ± 3.7
Time-To-Lift (s)	45.3 ± 2.8	33.2–59.0	48.0 ± 3.2	41.1 ± 4.7
Time-To-Doff (s)	7.2 ± 0.4	5.6–9.3	7.3 ± 0.6	7.2 ± 0.8

Note: For the system usability test, time-to-don, time-to-lift, time-to-doff, and usability scores were evaluated with 10 participants (6 novices, 4 expert users). For the usability score, participants were asked to answer ten questions, including the three in this table, from strongly disagree to strongly agree on a scale of 1–5. Data shown are mean and standard deviation, excluding range.

### Robustness to warehouse tasks: evaluation of controller performance and biomechanical efficacy in a real-world task simulation

**Fig. 4:**
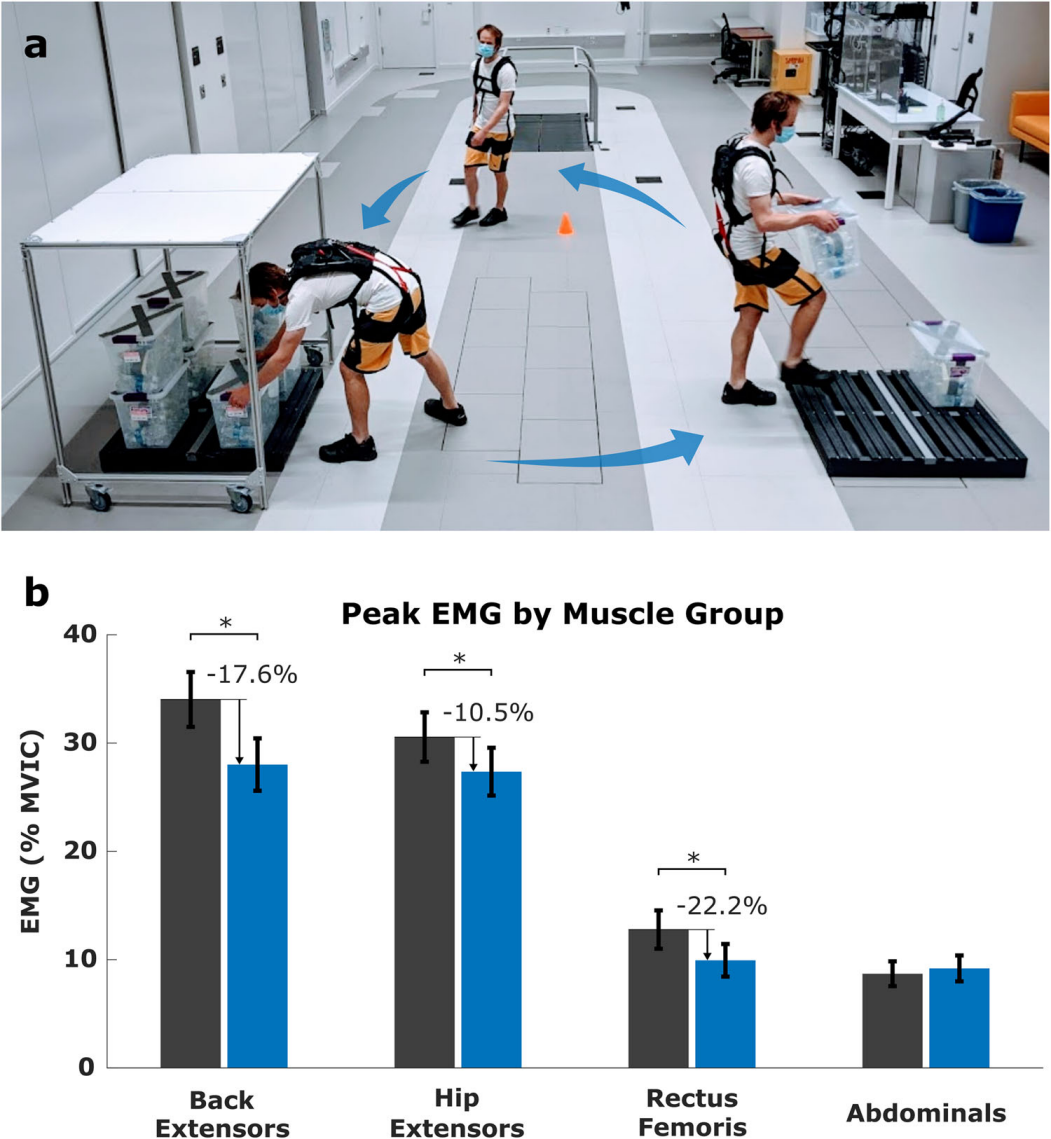
Order picking task. **a** Across an hour, participants completed 320 cycles, which included picking up a box from under a covered shelf, transferring the box to a pallet in an open space, and walking around a cone before returning to the covered pallet. **b** Peak electromyography (EMG) amplitudes normalized to maximum voluntary isometric contraction (MVIC) from three muscle groups decreased when lifting with exosuit assistance (blue bar) compared to lifting without (gray bar). Bar plot data show mean ± standard errors of data averaged across 14 (rectus femoris) or 15 participants. Individual data points are shown in Fig. S4. Significant differences (*P* < 0.01) to the no-suit condition are indicated by an *.

To test the controller performance and biomechanical efficacy of our active exosuit in a workplace environment, 15 participants performed a 1-h work simulation task on two separate days, once with and once without the exosuit in a randomized order (Fig. 4a; see details in Methods). Participants completed 320 cycles, which included picking up a box from under a covered shelf, transferring the box, building a pallet in an open space, and walking around a cone before returning to the covered pallet, for a total of two lifts per cycle (Movie S2). We measured trunk and thigh kinematics and peak and median muscle activities for back extensors, hip extensors, rectus femoris, and abdominals (details in the method section).

We found that our controller worked robustly with only 14 mistriggers (0.1%) out of 9600 lifts (15 participants × 640 lifts). All mistriggers occurred during picking up under a shelf, while no mistriggers occurred during pallet building lifts (Table S4). Moreover, all but one mistrigger occurred within one participant. To understand if the transition velocity range could explain controller robustness, we simulated four additional velocity thresholds (details in supplement) to show both mass position and velocity thresholds had an impact on the probability of mistriggers (Table S4) and only the 120° s-1 transition velocity was successful in reducing mistriggers below 1% for all mass orientations. Load cell data confirmed that the exosuit applied 18.6 ± 5.4 Nm of peak assistance across the variety of lift positions on the pallet, with minimal (0.33 ± 0.7 Nm) error (Table S5).

The exosuit assistance reduced peak EMG activity of the back extensor, hip extensor, and rectus femoris by 18%, 11%, and 22% compared to the no-suit condition, respectively (condition × muscle group *F*(3,3506) = 40.0, *p* < 0.001; Fig. 4b, Fig. S2 and Table 2). Similarly, median EMG activity of the back extensor, hip extensor, and rectus femoris were reduced by 20%, 13%, and 20% for the exosuit condition (Table S6). No condition effect was captured for the peak and median abdominal activity, suggesting no significant co-activation. These changes in muscle activity were consistent throughout the order picking test. Analysis of peak and median EMG amplitude at four 15-min epochs demonstrated no condition by epoch interactions (*p* > 0.05, Tables 2 and S6). However, epoch main effects captured both peak and median EMG amplitudes, which decreased across all muscle groups as the task progressed (Tables 2 and S6).

**Table 2:** Peak (90th percentile) EMG amplitudes (mean + SD) reported as % maximum voluntary isometric contraction (MVIC) during the picking task for 4 muscle groups at 4 epochs (time periods)

Condition	Back Extensors^h,k,a^ (% MVIC) (*N* = 15)	Hip extensors^k,a^ (%MVIC) (*N* = 15)	Rectus femoris^a^ (%MVIC) (*N* = 14)	Abdominals (%MVIC) (*N* = 15)
Time 0–15†‡✣				
No Suit	34.5 ± 10.3	33.8 ± 11.7	15.4 ± 8.6	8.8 ± 5.1
Exosuit	30.0 ± 10.8	30.4 ± 10.8	12.0 ± 6.6	10.8 ± 5.8
Time 15–30 †‡✣				
No Suit	33.7 ± 11.1	30.3 ± 10.7	12.8 ± 6.9	9.2 ± 4.8
Exosuit	28.0 ± 10.1	27.5 ± 10.0	10.0 ± 5.5	9.8 ± 5.2
Time 30–45				
No Suit	33.6 ± 10.9	29.1 ± 9.9	11.7 ± 6.9	8.2 ± 4.5
Exosuit	27.5 ± 9.6	26.3 ± 10.2	8.9 ± 6.3	8.5 ± 4.2
Time 45–60				
No Suit	34.3 ± 11.2	28.9 ± 10.0	11.2 ± 7.0	7.4 ± 3.8
Exosuit	26.5 ± 9.8	25.2 ± 10.5	7.9 ± 5.1	7.7 ± 3.9
Average (Time 0–60)				
No Suit	34.0 ± 10.8*	30.6 ± 10.8*	12.8 ± 7.6*	8.7 ± 4.6
Exosuit	28.0 ± 10.1	27.4 ± 10.5	9.9 ± 6.5	9.2 ± 5.0

Statistics	F-Score		probability	
Condition	F(1,3506) = 185.4		***p*** < **0.001**	
Epoch	F(3,3506) = 48.4		***p*** < **0.001**	
Mgroup	F(3,3506) = 2946.3		***p*** < **0.001**	
Cond*Epoch	F(3,3506) = 0.7		*p* = 0.549	
Cond*MGroup	F(3,3506) = 40.1		***p*** < **0.001**	
Epoch*MGroup	F(9,3506) = 2.5		***p*** = **0.007**	
Cond*Epoch*MGroup	F(9,3506) = 0.9		*p* = 0.565	

Note: *N* represents the number of participants with complete EMG data for a specific muscle group. Significant main effects and interactions are highlighted in bold, and trends are indicated by an underline. Significant muscle group by condition post-hocs (*) demonstrate when the no-suit condition is different from the exosuit condition within a specified muscle group (column) in the average row. Post hoc between muscle groups are denoted by superscript letters to show when the activity of the indicated site is higher than the back (b), hip (h) or knee (k) extensors, and the abdominals (a) in the condition (top) row. Significant epoch main effects are denoted by superscript symbols to show if there is higher activity than epoch 1(†), 2 (✣), 3 (‡) & 4 (✣) in the condition column. Other post hoc symbols are not included in the table.

Considering secondary outcome measures, the exosuit did not significantly alter the lifting style of users, but there was a trend towards participants adopting more squat-style lifts (Table S5). We did observe participants had slightly higher peak trunk flexion angles in the exosuit condition (100.4 ± 28.4°) than in the no-suit condition (99.8 ± 28.7°) (condition main: *F*(1,882) = 5.9, *p* = 0.015) (Table S5). Non-significant differences were acknowledged for peak trunk velocity and the time to complete a lifting or lowering task (Table S5).

## Discussion and conclusions

We developed and evaluated a soft active back exosuit suitable for assisting dynamic and varied activities commonly found in warehouse operations. The lightweight (2.7 kg) active system is easy to wear (average donning time 35.6 s) and minimally restrictive for lateral bending and twisting, common motions during order picking and pallet building tasks. An excellent usability test score highlighted that the device is intuitive and highly usable. An hour-long dynamic warehouse simulation demonstrated that our adaptive impedance controller is robust to common warehouse activities such as walking, lifting, picking up an item under a shelf, and building a pallet, with only 14 mistriggers out of 9600 lifts. Evaluating our device in a complex order picking task demonstrates our system successfully reduced peak and median back extensor muscle activity by 18-20% suggesting it has real-world potential in mitigating the risk of back injuries.

Despite our back exosuit delivering similar maximum assistance (30 Nm), our study achieved higher median EMG reductions (20%) than the 10.5% mean EMG reduction measured when deploying a rigid, passive device for an order-picking task^33^. Although different task demands and population characteristics can partially explain differences between these studies^3^, it is also worth considering that discrepancies may arise from variations in the system’s actuation strategy. While passive devices have shown impressive back EMG reductions for constrained squat, stoop, and asymmetric lifting tasks^9,35^, they often display diminished biomechanical efficacy in field-based studies when directly compared to results from the same device during constrained lifting tasks^20,33,36,37^. Interestingly, our system achieved slightly greater, 18% reductions in peak EMG amplitudes in our simulated work task, compared to 15–16% peak EMG reductions achieved during constrained lifting tasks^10,38^. It has been speculated that these differences reported for studies with passive devices are in part due to their limited ability to adapt to certain workplace demands^5,21,33^. Regarding movements involving less back flexion, passive back exos utilizing linear elastics provide lower peak assistance^10,11,39^. Consequently, this can lead to reduced mean EMG reduction when individuals lift stacked boxes that require less bending^40^. By utilizing a sine impedance approach (Fig. 2c), our exosuit delivered more than 80% of peak assistance over the wide range (53–127°) of trunk flexion angles^10^, which participants frequently passed through during the order picking task (Table S5). This broad high-assistance range of the adaptive impedance controller, though still providing diminished support for shallow bending below 53°, could explain how it consistently reduced back extensor EMG even when participants were tasked with lifting and lowering stacked (high) boxes (Tables S9 and S10). Moreover, passive back exos have been reported to apply 20–40% less torque during extension, where peak back extensor activity occurs^41^, due to mechanical hysteresis^42^. For our active exosuit, system hysteresis was not an issue as the low-level force controller tightly tracked the commanded forces with a RMSE of 0.33Nm (Fig. S3, Table S5). When accounting for these differences, our exosuit delivered 20-50% higher assistance during back extension compared to the passive device used by Motmans^33^, likely explaining why we achieved a higher reduction in median EMG amplitudes.

Unlike passive systems that are inherently robust, active systems offer the potential to adapt to uncontrolled dynamic tasks. However, the assistance needs to be applied robustly to avoid unhelpful forces that limit a user’s trust in the system and threaten the biomechanical efficacy and spinal stability^5,43^. Our adaptive impedance controller worked robustly during the order picking task, even though half of the lifts were performed underneath a shelf. These movements could increase the probability of inducing mistriggers (Table S4) when individuals had to engage in complex non-lifting motions to avoid obstacles while lifting. We intentionally used wide velocity thresholds during the transition phase to accommodate uncertainty from ambiguous small movements (Fig. 3b), minimizing mistriggers observed with lower velocity thresholds (Table S4). Taking this approach may lead to controller inefficiencies, to be discussed, but it is less sensitive to slight changes in movement direction or angular displacement compared to a near-binary approach employed in other studies^23,24^. This feature ensures a robust delivery of assistance, avoiding rapid switching between higher and lower assistance levels during small movements under the shelf (Fig. 3d). Although it is plausible that including additional sensors (e.g., detecting external load on the hands with EMG sensors or a pressure glove) could also result in active exos adapting to this task robustly, we opted to only utilize the motion sensors integrated within our exosuit to minimize usability challenges presented by donning additional sensors^5,21,26,27^.

In addition to reductions in peak and median back extensor EMG amplitudes, this study demonstrated exosuit assistance also reduced peak and median hip and knee extensor EMG amplitudes by 11–13% and 20–22%, respectively (Tables 2 and S6). Considering single muscle sites, these reductions were generally consistent for all muscles within the back, and hip extensors (Tables S7 and 8). While it was expected that hip extensor EMG amplitudes would decrease due to the assistive forces generated by the exosuit’s nylon ribbon cable spanning the hip (Fig. 1a)^10,22^, the reduction in knee extensor activity was somewhat unexpected. Studies have shown back exosuits can reduce knee extensor activity^3,40^, which could be explained by the line of action from exosuit assistance pulling the knee into extension. Alternatively, it was speculated that the exosuit may have influenced user kinematics. However, while most kinematic changes were not significant, there was a trend towards participants adopting a greater squat-style lift (Table S5), which would increase EMG amplitudes for knee extensors^9^. Ultimately, while the percent reduction in EMG amplitudes around the knee appears the largest when considering reduction in MVIC, the absolute benefit from the exosuit is lower compared to the % MVIC reduction observed for the back extensors and hip (Tables 2 and S6).

By monitoring EMG over the total duration of the order picking task, this experiment was able to demonstrate that exosuit effects remained consistent over 320 lifts. This finding suggests participants could adapt to back exosuit assistance early in the order picking task, as a previous study demonstrated motor adaptation to a wearable robot could improve the efficacy of the device over time^44^. An interesting finding was that peak and median EMG activity reduced as the task progressed (Tables 2 and S6). While fatigue during the palletizing task might have led to increased EMG activity^45,46^, the lack of increased EMG amplitudes indicates that the task was not highly fatiguing, likely explained by the relatively low mass lifted by participants and the walking recovery period between lifts. Despite no direct interactions with the exosuit, decreased EMG amplitudes over time suggest that participants adapted to the task. As the participants in this study were considered novice lifters, their adoption of expert lifting strategies, such as positioning boxes closer to the midline and scaling muscle activity to task demands, likely contributed to the observed EMG reductions^45,46^.

High system usability was a priority in the design of our back exosuit, as limited usability is a critical challenge hindering real-world adoption of back exos^3,6,7^. Multiple review papers point out that weight, ease of donning/doffing, and joint misalignment impact user acceptance and adoption in the field^7,8^. By carefully choosing the system requirements (30 Nm max assistance, integrating motion sensors needed for the controller, and soft force transmission mechanism), we were able to design a lightweight active device (2.7 kg) with minimal restriction (no restriction in lateral plane, 4.5% smaller RoM in frontal plane) that can be quickly donned (36.5 s). Although active systems might be thought of as more complicated, our adaptive impedance controller was considered both easy to use (4.7/5) and learn (4.9/5) (Table S3). Holistically, when evaluated by SUS, a usability test tool widely used for evaluating commonly used products, our exosuit achieved a high overall usability score (92.8), placing it in the 99th percentile^47^. However, our usability assessment did not have participants insert or remove batteries into the device, which might have changed the user’s perception of the device’s overall usability.

For active exos, there is a fundamental trade-off between system usability and biomechanical efficacy since higher assistance often requires a heavier actuator. Compared to the 26.5 ± 9.9% reduction reported by other untethered active back exos during constrained lifting, our 18-20% peak and median reduction in back extensor EMG might be viewed as moderate even though it is achieved in an hour-long simulated order picking task (Table S1)^3,4^. This is likely explained by our exosuit delivering lower peak assistance (30 Nm) compared to other active systems (38.1 ± 20.0 Nm). As a recent modeling study estimated that 30 Nm of assistance could reduce cumulative tissue damage by up to 70% if worn for an entire work shift^48^, we sought to reduce device weight as much as possible to increase intention to use^6^, while still achieving 30 Nm assistance. Surprisingly, while our system delivered lower assistance than other untethered active systems when normalizing our back extensor EMG reduction relative to overall system mass, our approach performed well compared to other systems (Table S1). However, a direct and fair comparison between devices is difficult, given differences in the time-varying actuation strategies, experimental setup, tasks, and normalization procedures between studies^10,21^.

There are a few limitations of the adaptive impedance approach. First, although the exosuit always applies assistance during flexion, the exosuit assists in lowering less than lifting in order to reduce movement restriction (Fig. 2b–d), at the cost of reduced biomechanical efficacy^10^. On occasions, a higher force may be desirable, such as when lowering a box to the ground, because the lumbar moment requirement is similar to lifting a box^41^. Prior work has addressed the importance of loading on assistance by integrating additional sensors like pressure gloves^42^. In this work, we prioritized minimizing the number of sensors required to maximize system usability. Future work will explore how to automatically detect loading information (holding a load or not, the mass of a load, etc.) and determine how forces should be adapted accordingly to maintain high usability. Another limitation of our adaptive impedance controller is the large transition window, necessitating the user to exceed the high extension velocity threshold for maximum assistance. This introduces a potential delay in delivering maximum assistance, which recent research suggests would provide less biomechanical benefit than delivering assistance more quickly^49^. Our controller framework could achieve similar outcomes by narrowing our transition velocity, but at the cost of more mistriggers (Table S4), potentially negatively impacting the perception of control^30^. Future work should focus on robust methods for quick transitions. Despite prioritizing perceptual improvements, our back exosuit significantly reduced peak and median back extensor activities by 18 and 20%. Third, while our method of calculating trunk flexion angle by subtracting thigh flexion angles effectively reduces exosuit assistance when walking, it comes at the cost of decreased device support for lunging lift (See supplementary notes). More sophisticated activity recognition algorithms might be able to assist these types of lifts with support comparable to lifts performed with symmetric thigh flexion. Lastly, the passive and inertial properties of our back exosuit can potentially modify participants’ biomechanics^50^. However, assuming a transparent (10 N assistance) exosuit experiment would impose a metabolic and biomechanical penalty^29,51^, we elected not to include this condition to demonstrate the exosuit with assistance provides biomechanical benefits compared to a no-suit condition. For these reasons, there is merit in conducting additional studies to understand whether sophisticated (faster-acting activity recognition algorithms) controller strategies can be deployed robustly in a complex work environment and yield biomechanical or perceptual improvements over our generic adaptive impedance controller strategy.

There are additional methodological limitations to the study. First, there is no measure of muscle forces or spinal compressive forces to understand the effects of applying exosuit forces in parallel with the spine^8^. Overall, the peak force applied by the exosuit (250 N) is small (3–8%) compared to the estimated peak spinal compression forces (3.5–6.5 KN) generated by biological muscles when an individual lifts a 10 kg mass off the floor^52,53^. Previous studies proposed that an external muscle architecture, which applies these small assistive forces with a larger moment arm than the user’s biological muscles, could reduce overall spinal compression by reducing muscle forces more than added assistive forces^16,39^. Consistent with this proposal, our study found an 18% reduction in peak back extensor muscle activity. Given our study observed minimal or non-significant changes in kinematics and abdominal co-activation, musculoskeletal models would likely estimate a reduction in muscular forces^54^. Second, although back exos can modify temporal features of EMG (Fig. S4)^55^, we could not analyze temporal components of EMG signals within each lift. This limitation arose from the fact that participants were free to choose their own movement strategies, which introduced considerable variability in the timing of their movements.

In conclusion, we developed and evaluated a back exosuit in a usability test and an hour-long order picking test. We showed that the back exosuit is highly usable and can robustly assist simulated common warehouse operations by offloading the back extensor muscles. Considering the demonstrated system usability and biomechanical efficacy, future studies should evaluate our back exosuit during field studies to probe whether this approach can indeed improve device usability and reduce the risk of long-term back injuries in the real world.

## Methods

### Soft exosuit and controller

#### Functional apparel components

Pivotal to this study was the inclusion of our exosuits. The functional apparel components of the exosuit consist of a backpack structure and two thigh wraps. The backpack houses the actuation unit and comfortably transmits the assistive force to the back via foam layered shoulder straps. The thigh wraps were constructed with lightweight, inextensible sailcloth layers with a high-friction inner interface to prevent slipping. The exosuit had adjustable shoulder and chest straps to accommodate various body sizes. Thigh wraps are adjustable using hook-and-loop fasteners (Velcro), laces, and a tensioning dial (L4, Boa Technology, Inc., CO, USA). A ribbon cable transmits forces between the actuator on the backpack structure and the top of the individual’s thigh wrap.

#### Actuation system and sensors

A cable-driven actuation system applies exosuit assistance. A brushless DC motor drives a pulley system that achieves torque amplification (4:1 ratio) and is directly connected to a spool wound with a one-inch ribbon cable. A hall-effect encoder measures motor position, and a load cell (LSB200, FUTEK Advanced Sensor Technology, Inc., CA, USA) placed in the actuation unit measures tensile forces on the ribbon. Three inertial measurement units (IMUs) (BNO085, CEVA, Inc, MD, USA) located on the backpack structure and the posterior part of the thigh wraps measure trunk and thigh kinematics. Two lithium-ion batteries (RRC2054, RRC power solutions GmbH, Germany) located at the top of the backpack structure are used in series to supply 28.8 V to the system allowing for 12 h of exosuit assistance when operating at 200 lifts per hour with 250 N peak force.

#### Controller unit

A custom controller unit with a 32-bit microcontroller unit (MCU; ATSAME70N21, Atmel Corp., CA, USA) generates force profile and performs force control at 1 kHz. Custom electrical boards with a 8-bit microprocessor units (PIC18F25K80, Microchip Technology, Inc., AZ, USA), an IMU, an analog digital converter, a motor driver (Gold Twitter, Elmo Motion Control, Ltd., Israel), and a controller area network (CAN) module are placed on the back panel and each thigh to read analog force signals from the load cell, as well as kinematic information (Euler angles, angular velocities, accelerations). The main MCU communicates with the distributed 8-bit microcontrollers and the motor driver through CAN communication protocol. All the algorithms were programmed in standard C language.

#### Relative angle

The force commands are generated based on the relative angle and angular velocity. As illustrated in Fig. 2a, the relative angle is defined as θ_rel_ = θ_trunk_ + 0.5 (θ_RT_ + θ_LT_) − 12° − 0.3 abs(θ_RT_ − θ_LT_), with θ_trunk_, θ_RT_, and θ_LT_ representing the flexion angles of the trunk, right thigh, and left thigh in the sagittal plane, respectively. Our rationale for this definition encompasses two principal objectives. Primarily, we aim to provide assistance for both squatting and stooping within a unified framework. This is realized by the relative angle’s capacity to encapsulate overall movement via quantifying the relative flexion between the trunk (θ_trunk_) and the thigh (*0.5* (θ_RT_ + θ_LT_)). Secondly, we intend to prevent from delivering assistance during walking, considering the potential disruption to users, as demonstrated by Poliero et al.^13^. This is accomplished by subtracting the fixed margin (12°) and the absolute difference between thigh flexion angles (abs(θ_RT_ − θ_LT_)), as these angles in the sagittal plane typically diverge during walking. This relative angle definition effectively makes the relative angle less than 0° during walking, resulting in the exosuit being transparent.

### Order picking test

#### Participants

For the order picking protocol, fifteen participants, eleven men and four women (31 ± 4 years old, 73 ± 12 kg, 172 ± 13 cm, with a BMI of 25 ± 5 kg m^−2^) volunteered (Table S11). Participants were screened to ensure they were sufficiently active and did not have health conditions that could interfere with their ability to perform the experiments, and they provided informed consent to a study approved by the Harvard Medical School’s Institutional Review Board (IRB18-0960). To note, no participant was involved in the design of the device. However, three of these individuals did participate in a similar developmental study in the last 3 months.

#### Experimental procedure and general study design

All participants attended a two-hour familiarization session and two formal experimental sessions separated by 5 ± 2 days. During the familiarization session, ten participants completed a frontal and transverse plane range of motion test (see supplement for details). Experimental conditions were analyzed in a repeated sample design. The order of task conditions was randomized using a counterbalanced Latin square. For all experimental sessions, participants were prepared for data collection, including the placement of three IMUs (MTi-3 AHRS, Xsens Technologies B.V., Enschede, the Netherlands) on the eight thoracic spinous process (T8) and the posterior aspect of the left and right thigh and sampled at 200 Hz. These three IMUs, separate from the IMUs integrated into the exosuit, were employed to gather participants’ motion data under both Exosuit and No Suit circumstances. EMG positioned over eight muscle sites from four muscle groups including the back extensors (lumbar longissimus and the thoracic and lumbar iliocostalis), trunk flexors (rectus abdominis and external obliques), hip extensors (gluteus maximus, biceps femoris), and the rectus femoris, were sampled at 2148 Hz using Duo wireless bioamplifiers and EMGWorks Software (Delsys Inc. Natick, MA, USA). EMG placement was followed by a series of maximum voluntary isometric contractions (MVIC) to normalize EMG signals^56^. Full data collection, data processing, and normalization are presented in the supplementary methods.

#### Order picking protocol

The participants performed a 1-h order picking task with or without the exosuit, defined by their Latin square order. The setup for the task involved participants lifting a 10 kg (43 × 28 × 32 cm) mass from a pallet situated below a 4 ft high covered rack, transferring the mass to an uncovered pallet positioned 8 feet away (Fig. 4a). Participants would walk unloaded to a cone 8 feet perpendicular to the shortest distance between the two pallets. No instructions were provided regarding style or speed. Some constraints were imposed to aid in the interpretation and to ensure consistency between the exosuit and no exosuit days. A completed pallet is composed of 8 boxes in a 2 × 2 × 2 stack. Contained with a column, boxes vary in depth (close and far) and height (high or low). Participants were instructed to work on one depth at a time, replacing the mass at the opposite depth. For example, lifting from the close high and replacing it far low. When a pallet was emptied, a researcher pushed the rack over the newly completed pallet. Thus, participants were obstructed when lifting the mass. Participants were encouraged to maintain a constant tempo, performing one box transfer every 12 s timed to a metronome. To handle the large quantity of data, participants were provided a 1–2 min break every 15 min to save data.

#### Data analysis

EMG signals were band-pass filtered (50–450 Hz)^57^, rectified and converted to a 6 Hz low-pass linear envelope that was amplitude normalized to peak activity measured during MVIC trials^58,59^. Exosuit load cell and IMU data were processed and corrected using a 2 Hz low pass filter in a Custom Matlab code (see supplementary methods). IMU data were utilized for event detection and as a primary outcome measure. Custom Matlab code (The Math WorksTM, Natick, MA, USA) was developed to threshold relative trunk flexion to indicate the beginning and end of a task for time-normalization.

Approximate peak EMG amplitudes were calculated using the amplitude probability distribution map (APDM)^37^. These measures were segmented during periods of lifting and lowering. Peak EMG was calculated as the 90th percentile EMG amplitude for each event. Median EMG was also calculated as a secondary outcome measure. To simplify data interpretation and to compare fairly to other lab groups, peak and median muscle activity was averaged across the 4 principal muscle groups (back extensors, abdominals, hip flexors, and the hip flexor (rectus femoris)) within each participant, condition, and task combination (see supplementary methods^60–63^). However, for completeness, EMG amplitudes at each muscle site (Tables S7 and 8) and EMG amplitude changes at each muscle group when lifting and lowering objects at various heights (Tables S9 and 10) are included in the supplementary material.

A secondary outcome measure of this study is peak (90th percentile) trunk angular displacement. Peak absolute trunk angular velocity was calculated for each lifting event (see supplement for details). For both measures peak lifting and lowering activity was averaged for each mass position and event contained within a 15 min epoch. IMU measures were also used as a tertiary outcome to calculate whether a participant performed a squat or stoop-style lift with the exosuit (see supplement for details). To explore temporal dynamics, the duration of lifting and lowering was measured (see supplement for details). Finally, peak load cell moment and the root mean squared error (RMSE) between the desired force command and load cell measurements were calculated to describe device performance (see supplement for details^64^). When available, peak trunk angular displacement was also calculated during the range of motion tasks.

#### Statistical analysis

Statistical analysis was performed using Linear Mixed Model (LMM) ANOVAs to test all hypotheses. Within each experiment, significance was Bonferroni corrected for pairwise comparisons for co-primary outcome measures. Secondary outcome measures had a conservative alpha (0.01) to prevent type-I error. Significant interaction and main effects were post-hoc tested using Tukey’s HSD. These models assume linearity and normality, violations of the assumptions were remedied using transformations suggested by Johnson’s test in Minitab 19 (Minitab LLC, State College, PA). Within the manuscript, only significant conditions (exosuit vs no exosuit) and main effects or interactions were expressed.

To ensure study power, the sample size was calculated for our primary outcome measure, exosuit differences in peak back extensor EMG amplitudes. Data from previous studies^9,10^ demonstrate large EMG amplitude differences (effect size: *d* = 1.08) between exosuit and no-exosuit conditions, and 14 participants would be required to detect condition differences with 80% power (*α* = 0.05). For the order picking tasks, the primary outcome measure was peak EMG amplitude measured from four muscle groups. A three-factor LMM ANOVA included the following factors: (i) Condition (2—exosuit and no-suit), (ii) Muscle Group (4), and (iii) Epoch (4–15-min time segments). Secondary outcomes, including median EMG amplitudes, were analyzed using the same three-factor LMM ANOVA. Additional secondary outcome measures, including IMU trunk flexion angle and angular velocity were analyzed in a 2 factor LMM: (i) Condition (2—exosuit and no-suit), and (ii) Epoch (4–15-min time segments). An identical analysis was used for tertiary outcome measures. For the RoM task, IMU trunk axial rotation and lateral flexion were compared between conditions using a paired *t*-test.

### Usability test

#### Participants

Ten healthy participants (Table S12) were screened and consented into the usability protocol (IRB18-0960). This experiment was separate from the order picking task, and only one participant was involved in both experiments. Of these participants, six were novices, and four were considered expert users, defined as members of this research group that used the back exosuit more than 5 times.

#### Protocol

For the system usability test, all participants were briefly introduced to the back exosuit, describing the goals of the device. A research assistant demonstrated how to properly don/doff and power the device on/off. Following the demonstration, the participants put on the exosuit and adjusted all straps by themselves with supervision to ensure a comfortable exosuit fit. After powering on the exosuit, participants tried various movements with the exosuit’s assistance, including walking, back flexion/extension, axial rotation, and lifting. Then, they turned off the device and unclipped the sternum straps and thigh wraps by themselves. After 1–2 trials, participants felt comfortable to move onto the timed donning and lifting task.

During the timed tasks, following a go signal, the participant retrieved a suit from a hanger and donned the suit as quickly and accurately as possible. Once all straps were closed, a research assistant recorded don time. Participants then powered on the suit, letting the ribbon fully calibrate, and lifted a box. During this part of the task, time-to-lift was defined as the time between the go signal and when the participant grasped the object. Finally, participants would indicate when they were about to doff the device. A research assistant recorded the time from this verbal cue until the suit was off the participant’s body. This task was repeated 5 times to average don, lift and doff time. Upon completion of this task, participants used a tablet to complete the system usability scale (SUS) converted into a Qualtrics Survey (Qualtrics XM, Provo, UT)^34^. Given the small number of participants involved in the usability study, these data are descriptive and were not included in a formal statistical analysis.

### Reporting summary

Further information on research design is available in the Nature Portfolio Reporting Summary linked to this article.

### Supplementary information

Supplementary materials

Description of Additional Supplementary Files

Supplementary Movie 1

Supplementary Movie 2

Reporting Summary

## Data Availability

The derived data that support the findings of this study are available from the corresponding author (C.J.W) upon reasonable request.

## Code availability

The code developed to calculate these derivations is available from the corresponding author C.J.W on request.
