# Supplementary material for: Lightweight active back exosuit reduces muscular effort during an hour-long order picking task
Source: Commun Eng. 2024 Feb 23;3:35. doi: 10.1038/s44172-024-00180-w (PMC10955849; doi:10.1038/s44172-024-00180-w)
Supplement: Supplementary file 1 — Supplementary materials [file 44172_2024_180_MOESM1_ESM.pdf]

Supplementary Information for

Lightweight active back exosuit reduces muscular effort during an hour-long order picking task

Jinwon Chung *et al.*

*Corresponding author. Email: walsh@seas.harvard.edu

This PDF file includes:

1.0 Supplementary Methods
2.0 Supplementary Notes
3.0 Supplementary Figures S1 to S4
4.0 Supplementary Tables S1 to S12

Other Supplementary Materials for this manuscript include the following:

Movies S1 to S2

1.0 Supplementary Methods

Exosuit components

The exosuit is composed of the back panel and the thigh wraps. The entire system weighs 2,715 g, and it breaks down to thigh wraps (356 g), batteries (462 g), and the back panel assembly (1,897 g), including the actuation unit, the controller unit, the shoulder straps, and chest straps (Fig. 1). The size of the back panel is about 250 mm, 360 mm, 85 mm in width, length, and thickness, respectively. The length of the thigh wrap can be adjusted from 650 mm to 720 mm to accommodate various sizes of the thigh, and a 150 mm Velcro section enables attachment of the thigh wrap. The back panel is anchored to the torso of the user by shoulder straps, which can be adjusted for a wide range of body sizes using webbing straps and ladder locks. The chest straps adjust the distance between two shoulder straps so that the shoulder straps do not cut through the armpits of users, which would make them uncomfortable. The actuation unit includes a pulley system, a timing belt, a spool, and a ribbon cable. A brushless DC motor (U-type BLDC motor, T-motor, Jiangxi, China) drives a 4:1 pulley system with a timing belt to achieve torque amplification. The pulley is directly connected to a spool, which pulls the red ribbon and applies force between the torso and the thighs through the back panel and the thigh wraps (Fig. 1, main manuscript). Three IMU sensors are housed within the actuation unit, and the thigh wraps are used to measure the trunk and thigh kinematics of the user.

Recruitment

Participants were recruited through internal advertisements and word-of-mouth engagement. All participants included in experiments one and two were screened, ensuring they engaged in moderate physical activity more than 3 hours a week and were excluded if they reported a recent (<3 months) history of low back pain or any musculoskeletal or neurological conditions that could interfere with their ability to perform the experiments. Consistent with Helsinki's guidelines, all participants provided informed consent to a study approved by the Harvard Longwood Faculty of Medicine Institutional Review Board (IRB18-0960).

Order picking experiment: Participants

Fifteen participants volunteered for this protocol. Self-reporting sex: this study had eleven men and four women. The general demographics of the participants were an age of 31±4 years old, a mass of 73±12 kg, a height of 172±17cm, and a BMI of 25±5 kg/m^2 (Table S11).

Order picking experiment: Range of motion task

Ten of the order picking participants (6 men, 4 women, age 31±4, mass 73±14, height 176±11, and BMI 23±3 kg/m^2) completed a range of motion protocol 5 days prior to their participation in the order picking task. For these tasks, trunk motion was measured using the T8 IMU. Participants performed a maximum lateral bend and axial rotation task timed to a 50 BPM metronome. Starting from a comfortable standing position over two beats for each event, participants would rotate or bend to the right, hold that position, return to neutral, hold that

position, rotate or bend to the left, hold that position, and return to neutral. Participants repeated this cycle 3 times, both with and without the exosuit implemented in a random order.

Order picking experiment: Maximum voluntary isometric contractions (MVIC)

To normalize electromyography signals, participants performed three maximum voluntary isometric contractions (MVIC) [56,58,59]. For all normalization exercises, participants were secured to a rigid structure and asked to practice 1-2 warm-up contractions. Afterwards, the participants performed 3 repetitions of a maximum contraction where they were instructed to pull against non-elastic straps as hard as possible for 3 seconds, with 1-2 minutes of rest between trials to minimize the effects of fatigue [10].

To normalize the trunk flexors, participants sat comfortably on a chair with their trunk at a 90° angle. Participants were secured to the chair using a chest harness and performed a maximal effort crunch into the chest harness. Normalization of the hip and knee flexors were also performed seated. Using a padded cuff around their ankle, a strap was tensioned so the participant's knee was in 70-80° of flexion when the participant attempted to extend their knee maximally. Participants were positioned on a Roman chair to normalize the trunk and hip extensors. Participants assumed a prone position on the chair with their shanks under a posterior bar and their pelvis (anterior superior iliac spine) aligned to the edge of the chair. A torso harness secured the participant to the base of the chair using a strap designed to keep the participant's trunk parallel to their thigh during maximal back extension [10].

Order picking experiment: Data collection and analysis

Inertial motion unit and movement segmentation

An external IMU harness with three inertial measurement units (IMUs, MTi-3 AHRS, Xsens Technologies B.V., Enschede, the Netherlands) was used to capture participants' kinematics for both the no-suit and the exosuit conditions. The IMUs were positioned on a variety of anatomical landmarks by directly securing them to the participants' skin using a combination of adhesives (adhesive spray, double-sided tape, and cover roll tape) to prevent undesired motion. A single IMU was positioned on the eight thoracic spinous process to characterize torso angle. To characterize thigh angle, two additional sensors were placed bilaterally on the posterior aspect of the left and right thigh, approximately in line with the middle of the gluteal fold and popliteal fossa [10].

Prior to performing movement tasks, the IMU sensors were calibrated around the participants' neutral posture, acting as a reference zero angle for the subsequent conditions. Following calibration, roll kinematics (Flexion-extension) from each IMU (Euler angles) were directly sampled and saved at 1KHz using an 8-bit microprocessing unit (PIC18F25K80, Microchip Technology, Inc., AZ, USA) and an onboard flash memory card (SDSQUNC-032G-AN6IA, Scandisk, CA, USA). IMU angular data were post-processed using custom Matlab code and

corrected using a zero-lag 4th order 2 Hz low pass filter. Relative trunk angle was calculated using a subtraction method between T8 and the average of both the left and right thighs [10].

An event detection algorithm was developed using relative trunk angle in the sagittal plane to segment lifting events using custom Matlab code. The beginning of any lift or lower event was defined as the time that trunk flexion angle exceeded 30° neutral for at least 20 ms. The end of each lift and lower was defined as when the trunk angle became less than 30° of trunk flexion for at least 20 ms. Event algorithms were verified using visual inspection. Because participants lifted under a rack, it was common that these events included periods of carrying. Using a quadratic spline interpolation algorithm, these events were used to time-normalize all load cell, kinematic (IMU), and EMG signals over 1000 data points (0-100%). All time-varying data were synchronized with a common signal generated by Qualisys Track Manager (Version 2020.2, QualisysTM, Goteborg, Sweden).

As a primary outcome measure, trunk angle was calculated using time normalized T8 and thigh IMU signals. For each lifting and lowering event, peak trunk angular displacement was calculated by using the amplitude probability distribution map (APDM) to calculate the 90th percentile data point from the time normalized signal. Trunk angle was averaged across each lifting and lowering event within each 15 minute epoch.

As a secondary outcome, trunk angular velocity was calculated as the 90th percentile of the absolute trunk velocity for each lifting and lowering event. Each lifting event was classified as a squat or a stoop using IMU data and a custom algorithm to quantify lifting style. Using a ratio between torso (T8) flexion angle and the average (left and right) thigh flexion angle at the time of maximum relative trunk angle, lifts were described as a stoop if the contribution of thigh flexion angle to a lifting was less than 33%. Otherwise, the lifting posture was classified as a squat. The classification accuracy of this algorithm was developed during constrained lifting tasks with 99.15% accuracy. For the order picking task, IMU data were analyzed for each 15 minute block, to determine the % of stoop lifts performed by participants in the exosuit and no-exosuit conditions.

Electromyography

Following standard skin preparation, bar surface electrodes (10 mm interelectrode distance) were positioned over 8 muscle sites from 4 muscle groups using standardized guidelines and minor adjustments based on palpation [10]. Muscle sites for the back extensors included the thoracic (T95)[54] and lumbar erector spinae iliocostalis (L16) (5 cm lateral to the 9th thoracic and 1st lumbar spinous process, respectively) and the erector spinae lumbar longissimus (L33) (3 cm lateral to the 3rd lumbar spinous process). For the trunk flexors, signals were monitored from the upper rectus abdominis (URA) (3 cm lateral to the linea alba) and the middle external obliques (EO) (15 cm lateral to the umbilicus oriented 45° to the linea alba).[58, 59] SENIAM guidelines were used to position the hip extensors (gluteus maximus (GM) and biceps femoris (BF)) and the knee extensor (rectus femoris (RF)). To prevent motion artifacts and keep electrodes from

moving during the order picking task, EMG bar electrodes were secured to the skin using double-sided, and cover roll tape was affixed to the skin overtop of the EMG electrodes. To protect the electrodes further, a fabric foam belt was used to cover all abdominal and back electrodes. EMG signals were amplified, digitized (2148 Hz), and filtered (Hardware band-pass 20-450 Hz) using a series of Duo wireless bioamplifiers and EMGWorks Software (Delsys Inc., Natick, MA).

EMG signals were digitally filtered using a fourth order, 50-450 Hz zero-lag Butterworth Filter to correct for signal drift and motion artifact [57, 59]. Corrected signals were rectified and converted to a 6 Hz low-pass linear envelope [10, 38]. For each muscle, the time normalized (0-100%) linear envelope signal was amplitude normalized to the peak EMG captured during the MVIC tasks [56]. For each muscle, the EMG signal amplitude was sorted using an APDM approach. 90th percentile (approximate peak) EMG amplitudes were calculated for each lifting and lowering event.[37] As a secondary outcome measure, 50th percentile (median) EMG amplitudes were also calculated. We chose the APDM methodology as it is robust to outliers that could occur in the EMG signal arising from uncharacterized perturbations within a single lift or lower cycle. This approach offers an additional advantage by not necessitating temporal consistency, making it well-suited for electromyography analysis in less constrained settings. In our specific case, it effectively handles the temporal variability observed in both the participants' kinematic and electromyography signals during our freestyle order-picking task, as depicted in Figure S3 [37]. EMG signals were further processed to remove outliers defined as 90th percent amplitudes that were 4x higher the median and standard deviation of 90th percentile signals calculated within the same participant, muscle, condition, lifting or lowering activity and mass position within a 15 minute epoch. If EMG signals were lost from a specific muscle during data collection, no attempt was made to reapply the electrode, and the EMG signal was removed from statistical analysis across all conditions and epochs. For each muscle and participant, 90th percentile EMG amplitudes were averaged across each lifting and lowering event for a specific condition, 15-minute epoch, and box position across the 15-minute functional task. Thus, the entire functional task had peak and median lowering and lifting amplitude representing 4 15-minute epochs.

Data from each muscle site was categorized into 4 principal muscle groups (back extensors, hip extensors, hip flexors, and the abdominals) to improve the interpretation of EMG signals. Within a specific participant, condition, epoch, mass position, and lift or lower event peak and median EMG amplitudes were averaged across the relevant muscles contained in a muscle group that was compared for statistical analysis (Table 2 & S6).

For completeness, peak and median activity from each muscle site can be found in Tables S7 & S8. EMG amplitudes from each muscle site were analyzed using a three-factor LMM ANOVA that included the following factors: i) condition (2 - exosuit and no-suit), ii) muscle site (8), and iii) epoch (4 - 15-minute time segments). Finally, while not analyzed statistically, the breakdown

of peak and median muscle group activity for lifting and lowering activities for all mass positions are included in Tables S9 & 10.

Order picking experiment: Calculation of the suit moment

The exosuit moment was calculated using load cell measured tensile forces (See Methods Section) and custom Matlab Code for each lifting and lowering event [38]. To calculate suit moment contribution, time-normalized tensile force was converted to a rotational moment acting around the lumbar L5/S1 joint center with a constant moment arm length of 0.12m when considering flesh margin, suggesting the exosuit could produce a peak back extensor moment of 30 Nm [38, 60].

The peak (90th percentile) load cell moment was calculated as a tertiary outcome measure for each lifting and lowering event. Each participant's 90th percentile load cell moment was averaged across each lifting and lowering event for a specific condition, 15-minute epoch, and box position. To characterize the low-level controller of the exosuit, Root Mean Squared Error (RMSE) was calculated for each time point between the desired force command and measured load cell moment (Fig. S2). These data are presented in Table S5.

Order picking experiment: Binary controller simulation for Mistrigger Analysis

To understand the effect of our transition window during the order picking task, we simulated the force profile that would have been generated by the adaptive impedance controller with various transition windows (±120°/s, ±90°/s, ±60°/s, ±30°/s, and ±0°/s) using IMU data collected during the order picking protocol. Our ±0°/s transition window was considered a binary controller in the main text for this analysis. The simulated exosuit force command was analyzed for each lifting and lowering event seeking for mistriggers defined as if the force profile switched between lowering and lifting states more than once for a single lift. For each transition window, the percentage of mistriggers was calculated for each participant and mass position. These data were entered into a two-factor LMM ANOVA for threshold (5), and mass position (5).

2.0 Supplementary Notes

Order picking experiment: Mistrigger Analysis

This simulation analysis revealed controllers were more likely to result in mistriggers as the transition velocity range narrowed (Table S4). We also found that the likelihood of a mistrigger depended on box location and shelf coverage (Table S4). While narrow transition windows were less likely to mistrigger when stabilizing the pallet in open spaces (3.7-45%), multiple mistriggers occurred when participants reached for objects in tight spaces such as the back top quadrant of the covered pallet (15.9-97.8%). Considering the interaction with mass location, only the 120°/s transition window successfully reduced the probability of mistriggers to less than 1% for all mass locations (Table S4).

Influence of relative angle on assistance

The relative angle is defined in a way that the controller commands minimal pretension (10N) during walking and carrying so as to not hinder movement (Method - *Relative angle*). Consistent with our design, the controller commanded 11.90 ± 0.09N of force during walking and was consistent with the measured applied cable forces of 11.79 ± 0.09N (Fig. S2).

Notably, the relative angle has subtle implications on force commands in a few scenarios. The subtraction of abs (θ_{RT} - θ_{LT}) inherent to the relative angle calculation leads to diminished assistance during lunging, in contrast to the support provided during squatting and stooping. However, participants did not explicitly perceive this nuanced effect. Another intriguing scenario arises when a user walks while bending their back. In such instances, the controller generates forces exceeding pretension levels due to the trunk flexion angle (θ_{trunk}), potentially causing disruptive assistance. However, occurrences of this movement remained infrequent throughout the experiment, and participants did not report discomfort associated with this phenomenon.

Constrained movement test session prior to the order picking protocol

The order picking protocol presented in this manuscript was developed as part of a comprehensive three-session experiment. In addition to the order picking protocol, there was a separate constrained movement test session, where participants performed a series of controlled reaching and lifting tasks, and the findings from these tests were documented in a separate manuscript [10]. It is worth noting that participants always completed the constrained movement test before engaging in order picking sessions. Even though the constrained movement test was conducted 5±2 days before, it could allow participants to become familiar with the device, potentially enhancing the effectiveness of the exosuit forces as participants adapted to them [44].

Comparison with other active back exos

Current review papers do not provide enough information for device weight, max applied torque, and back extensor EMG reduction during lifting tasks. To understand the performance of our back exosuit, we conducted a brief comparison between our device and other published untethered active back exos that can be potentially deployed in the field in Table S1.

Pros and cons of using soft force transmission in terms of low-level force controller performance

Our device is classified as a soft exosuit as the device that applies assistance to the user via a flexible ribbon cable instead of rigid frames. The primary advantage we sought in employing a soft force transmission mechanism was to minimize the potential for movement restriction or joint misalignment that can arise with rigid transmission mechanisms [5]. However, designing the low-level force controller using this soft force transmission mechanism was a challenge, primarily due to the flexibility and deformation of the ribbon cable. The ribbon cable sometimes came into contact with various parts of the hardware, including the back panel and side wall of the spool. These interactions led to constant changes in friction and system dynamics. To account

for these variations, we had to develop a force controller that maintained stability across a wide range of conditions. Achieving this stability required reducing the controller gains and compromising force tracking performance to some extent.

Despite these challenges, we were able to achieve a relatively low force tracking error (0.33N, 1.1% of peak assistance torque), as demonstrated in Figure S2 and Table S5, as a result of the utilization of a high-frequency real-time force controller loop operating at 1kHz, coupled with the 20kHz current controller loop facilitated by the motor driver. However, these results do not suggest any specific advantages of a soft force transmission regarding low-level controller performance.

3.0 Supplementary Figures

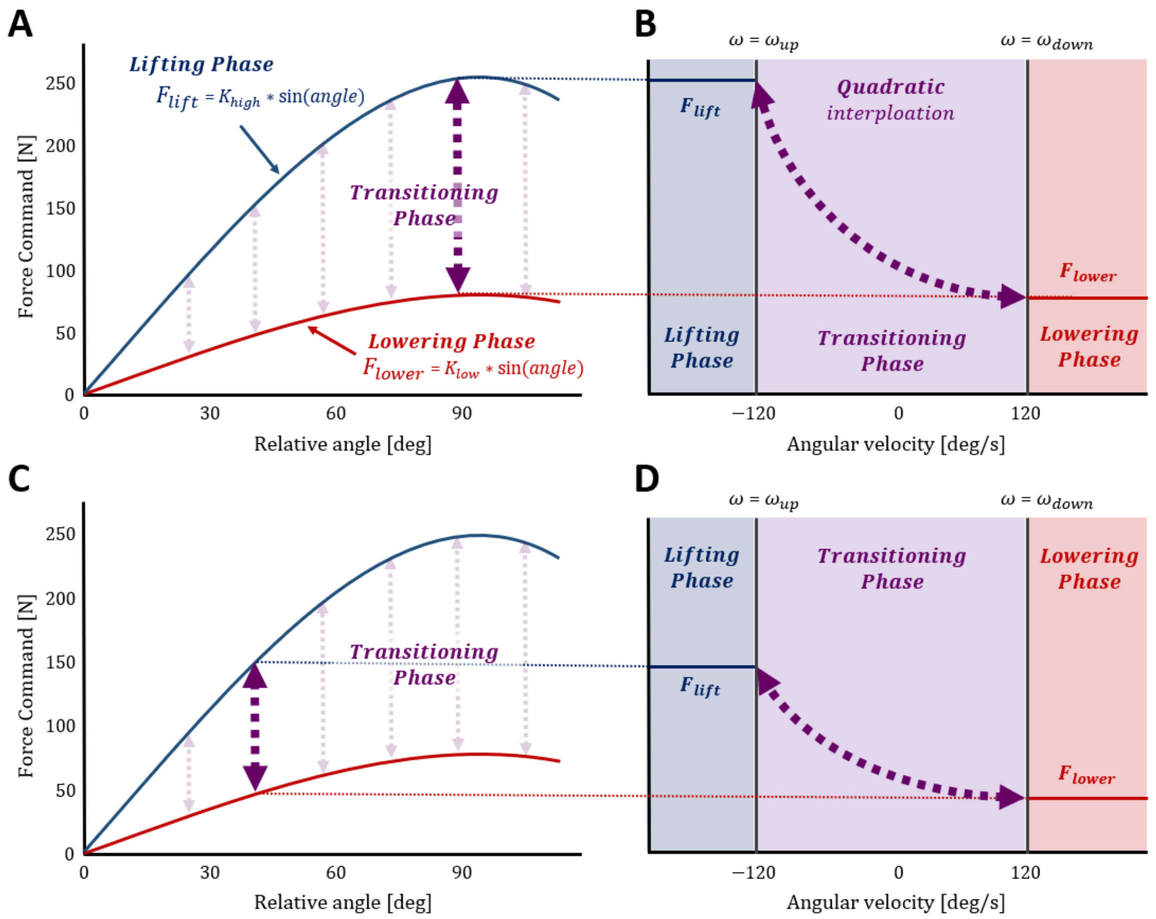

Fig S1. Example of force command at two different relative angles. Exosuit assistance is dependent on trunk relative angle (subplot A & C) and trunk angular velocity (w) (subplot B & D). These hypothetical data demonstrate at a relative trunk angle of 90°, A) maximum lifting (blue line) and minimum lowering assistance (red line) can be determined using the indicated equations, B) the slope of the transition phase (purple line) is determined by a quadratic interpolation at a specific trunk angle. Comparing example force commands for different bending depths, this figure demonstrates how assistance provided at 90° is substantially higher than the C) maximum lifting (blue line), minimum lowering assistance (red line), D) than a transition (purple line) that would be achieved if the trunk were to transition at 40°.

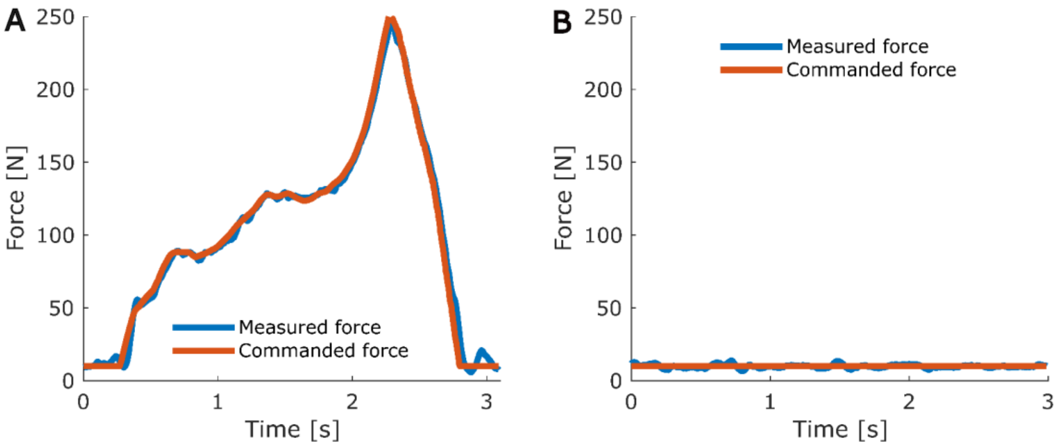

Fig S2. Representative force tracking profiles comparing the ideal force command (orange line) and the measured force command (blue line) measured during A) the lift cycle and B) the walking phase from the order picking task. It demonstrates the minimal error between commanded and measured force and how the exosuit B) applied minimal (~11N) assistance during walking.

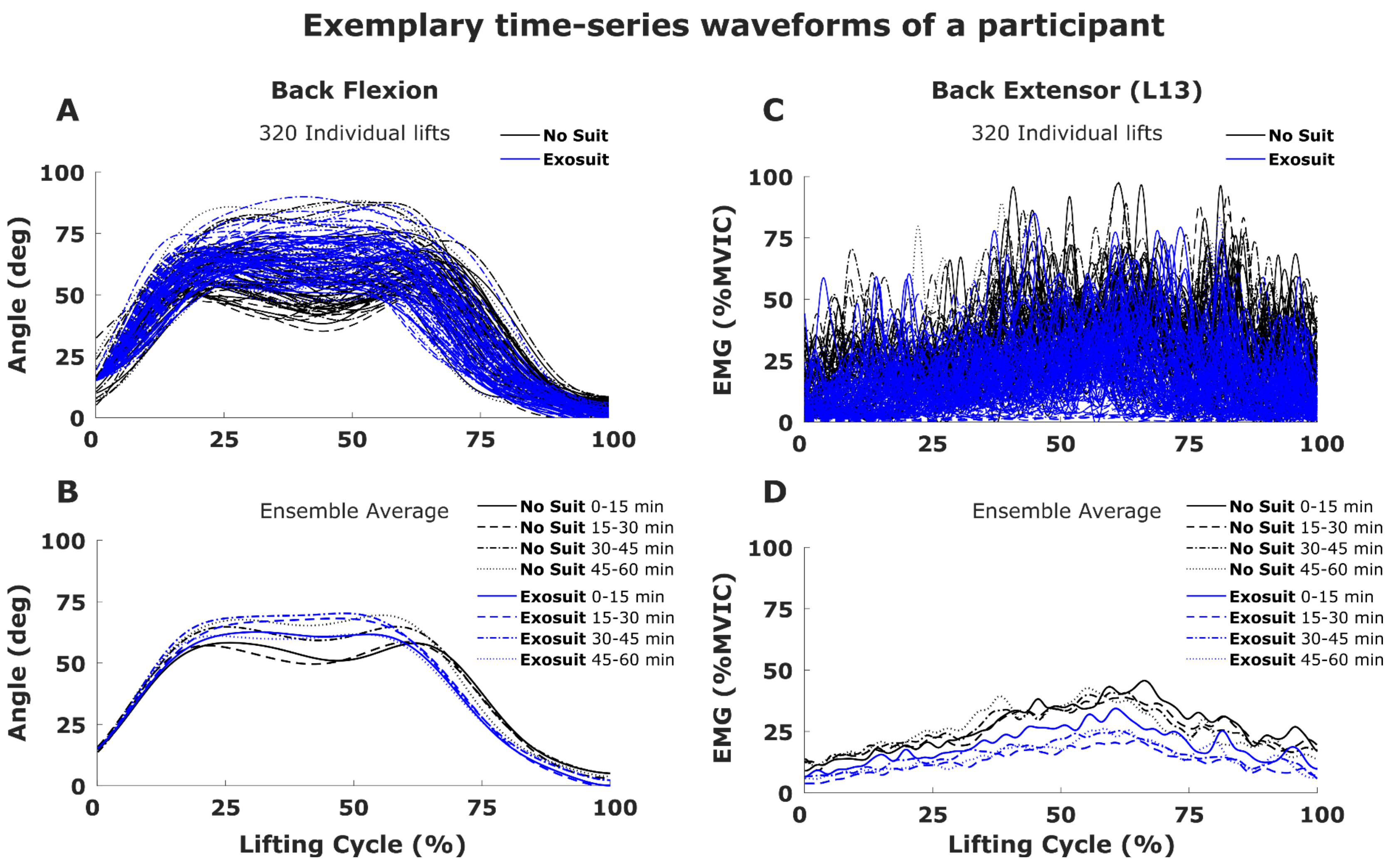

Fig S3. Exemplary linear envelope temporal waveform of one participant completing multiple lifting repetitions with a mass position in the far-high quadrant of the pallet. Subplot (A & C) captures the considerable kinematic (A) and electromyographic (C) spatial-temporal complexity for lifting repetitions within a participant for both the exosuit (blue line) and no-exosuit (black line) conditions. Although ensemble averages of trunk

flexion angles (B) and back extensor activity (D) can be produced over the numerous repetitions every 15 minutes (different line styles), they might not capture the period of peak EMG activity that can coincide when the participant begins to grasp and lifts the 10kg mass on the pallet.

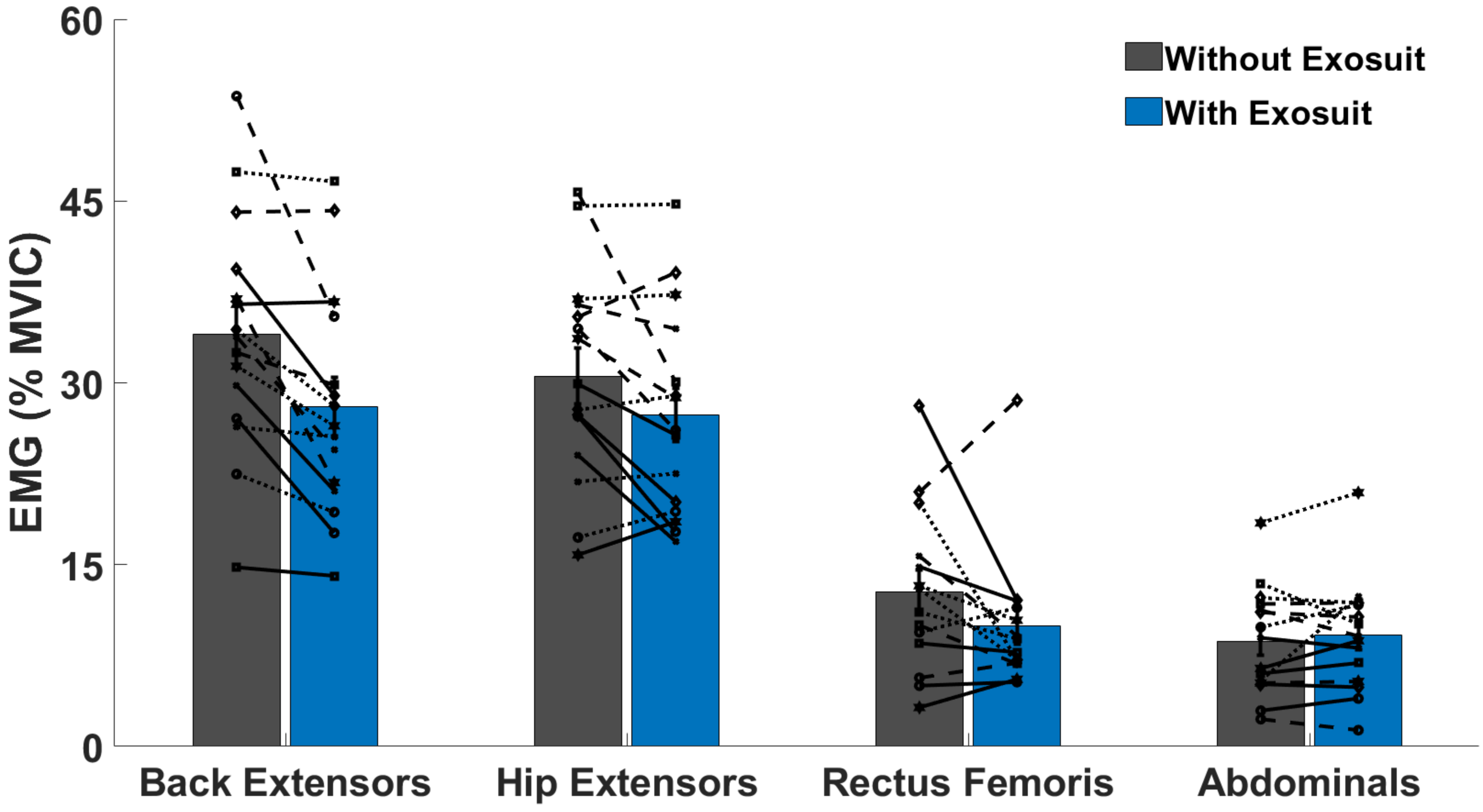

Fig S4. Individual Data Points of 90th Percentile EMG Amplitudes During the Palletizing Task. Peak electromyography (EMG) amplitudes normalized to maximum voluntary isometric contraction (MVIC) from four muscle groups when lifting with exosuit assistance (blue bar) compared to lifting without (grey bar). Bar plot data show mean ± standard errors of data averaged across 14 (rectus femoris) or 15 participants (all other muscle groups). Unique symbols and line styles represent data points from separate participants over their corresponding exosuit condition.

4.0 Supplementary Tables

Table S1. Active untethered back exos in the field.

Publication	Device name	Weight (kg)	Max applied torque (Nm)	Torque density (Nm kg-1)	Back extensor EMG reduction (%)	Back extensor EMG reduction/weight (% kg-1)
Ko et al. *[61]*	H-wex	4.5	45	10.0	p=17.0	3.8
Toxiri et al. *[26]*	Robomate	11.6	40	3.4	p=31.5	2.7
Huysamen et al. *[52]*	Robomate	11.6	N/A	N/A	p=13.5	1.2
Zhang et al. *[27]*	Lower back robotic exo	11.2	70	6.3	i=33.9	3.0
Glinski et al. *[63]*	HAL lumbar support	2.9	15	5.2	i=12.2	4.2
Yong et al. *[64]*	SiAT	5	N/A	N/A	i=30.7	6.1
Lanotte et al. *[28]*	APO	8	30	3.8	i=32.9	4.1
Poliero et al. *[13]*	XoTrunk	6.5	52	8.0	p=35, m=43	6.2
Li et al. *[23]*	ABX	6.4	14.8	2.3	r=26.9	4.2
**This paper**	**Back exosuit**	**2.7**	**30**	**11.1**	**p=18, m=20**	**6.7**

Torque information was gathered from the publication. When papers did not report max applied torque, values were calculated based on citations within the papers or from figures. EMG reductions were averaged across mass and relevant back muscles, and the letters p, m, i, or r included before % reduction refers to whether the data point was peak, mean, integrated, or root mean square, respectively.

Table S2. Maximum trunk angular displacement (Mean+SD) was calculated from participants (N=10) who completed the max range of motion task.

Condition	Axial Rotation (°)	Lateral Flexion (°)
NS	127.8 ± 30.2	59.2 ± 12.5
AS	123.5 ± 31.3	56.2 ± 10.7
Statistical Results		
T-Score	T(9)=3.14	T(9)=0.7
p-value	p=0.012	p=0.503

Significant t-tests are highlighted in bold, and trends are underlined. Data with a superscript identify a significant condition main effect (p<0.01) when compared to the no-suit(*) condition.

Table S3. System usability scale questions and scores. The system usability scale consists of 10 questions. Participants are asked to answer these questions from strongly disagree to strongly agree on a scale of 1 to 5. Data is displayed as mean and standard deviation except for the range column.

Questions	Overall (N=10)	Range (N=10)	Expert (N=4)	Novice (N=6)
I would like to use the exosuit device often	4.2±0.8	3-5	4.0±1.5	4.3±0.5
I think the exosuit is complex to use	1.2±0.4	1-2	1.3±0.5	1.2±0.4
I think the exosuit is easy to use	4.7±0.5	4-5	4.5±0.6	4.8±0.4
I require technical assistance to use the exosuit	1.1±0.3	1-2	1.3±0.5	1.0±0.0
I think the functionalities of the exosuit are well integrated	4.9±0.3	4-5	5.0±0.0	4.8±0.4
I think the functionalities of the exosuit are not consistent	1.1±0.3	1-2	1.0±0.0	1.2±0.4
I think most users can quickly learn to use the exosuit	4.9±0.3	4-5	5.0±0.0	4.8±0.4
I think most users have difficulties to use the exosuit	1.5±0.5	1-2	1.5±0.6	1.5±0.5
I am confident when using the exosuit	4.8±0.4	4-5	5.0±0.0	4.7±0.5
I need to learn more background information of the exosuit before use	1.5±0.7	1-3	1.5±1.0	1.5±0.5

Table S4. Percentage of simulated mistriggers during an order picking task based on mass location and the influence of a wide transition window.

Transition Windows	Open space [b,c,d,e]	Front-Top [c,d,e]	Front-Bottom [d,e]	Back-Top	Back-Bottom	Overall
0 °/s	45.5%	68.2%	72.1%	97.8%	92.6%	64.1% *†‡+
±30°/s	14.5%	25.5%	24.0%	61.1%	39.0%	25.9% *†‡
±60°/s	3.7%	3.0%	4.2%	15.9%	8.9%	5.9% *†
±90°/s	0.7%	0.8%	1.2%	4.0%	3.1%	1.5%
±120°/s	0.0%	0.3%	0.3%	0.3%	0.3%	0.1%

Statistics (Main Effect or Interaction)	F-Score	P-Value
Transition Threshold	F(4,336)=415.4	**p<0.001**
Mass Position	F(4,366)=33.8	**p<0.001**
Threshold * Position	F(16,366)=8.2	**p<0.001**

Significant main effects and interactions are highlighted in bold. Significant post-hocs for between transition thresholds use superscript symbols to demonstrate differences compared to 120 (*), 90 (†), 60 (‡), and 30 (+). Post hoc between mass position is denoted by superscript letters to denote higher miss triggering of the indicated area when compared to lifting an object positioned in an a) open space, or the: b) front top, c) front bottom, d) back top, and e) back bottom area of the rack. Interaction post-hocs are not displayed in the table.

Table S5. Trunk Kinematics, lifting style, lift time, and peak load cell measured suit assistance (Mean+SD) calculated from all participants (N=15) during the palletizing task at 4 epochs (time periods)

Condition	Trunk Angle (°)	Trunk Speed (° s-1)	Style (% Stoop)	Lift Time (s)	Peak Assist (Nm)	RMSE (Nm)
Lifting 0-15 minutes						
NS	102.5 ± 28.4	92.6 ± 31.1	73.9 ± 28.8	3.01 ± 0.67	0.0 ± 0.0	0.0 ± 0.0
AS	102.0 ± 28.2	93.2 ± 30.7	68.4 ± 27.2	3.03 ± 0.76	19.0 ± 5.3	0.33 ± 0.07
Lifting 15-30 minutes						
NS	98.7 ± 28.1	92.7 ± 30.2	75.6 ± 29.2	2.96 ± 0.68	0.0 ± 0.0	0.0 ± 0.0
AS	99.6 ± 28.2	93.5 ± 31.4	73.4 ± 26.0	3.00 ± 0.76	18.6 ± 5.5	0.33 ± 0.07
Lifting 30-45 minutes						
NS	97.9 ± 29.4	92.6 ± 30.5	78.4 ± 29.1	2.98 ± 0.71	0.0 ± 0.0	0.0 ± 0.0
AS	100.3 ± 28.9	94.1 ± 32.4	70.4 ± 25.2	2.97 ± 0.77	18.5 ± 5.5	0.32 ± 0.07
Lifting 45-60 minutes						
NS	100.1 ± 29.0	93.6 ± 30.9	75.4 ± 30.4	2.97 ± 0.70	0.0 ± 0.0	0.0 ± 0.0
AS	99.9 ± 28.7	93.5 ± 31.8	73.9 ± 25.3	2.97 ± 0.78	18.3 ± 5.4	0.32 ± 0.07
Average (Time 0-60)						
NS	99.7 ± 28.7	92.9 ± 30.7	75.8 ± 28.7	2.98 ± 0.70	0.0 ± 0.0	0.0 ± 0.0
AS	100.4 ± 28.4*	93.6 ± 31.6	71.6 ± 25.4	3.00 ± 0.76	18.6 ± 5.4*	0.33 ± 0.07*

Statistics						
Condition	F(1,882)=5.9<br>**p=0.015**	F(1,882)=1.0<br>p=0.311	F(1,98)=5.7<br>p=0.019	F(1,882)=0.8<br>p=0.366	F(1,822)=4.3^10^4<br>**p<0.001**	F(1,822)=3.5^10^4<br>**p<0.001**
Epoch	F(3,882)=0.5<br>p=0.674	F(3,822)=0.2<br>p=0.920	F(3,98)=0.9<br>p=0.442	F(3,822)=1.6<br>p=0.180	F(3,822)=3.0<br>p=0.031	F(3,822)=2.1<br>p=0.096
Cond*Epoch	F(3,882)=0.2<br>p=0.894	F(3,822)=0.8<br>p=0.841	F(3,98)=0.7<br>p=0.540	F(3,822)=0.5<br>p=0.702	F(3,822)=3.0<br>p=0.031	F(3,822)=2.1<br>p=0.096

Significant main effects or interactions are identified in **bold**, with trends indicated by an underline. Post hoc differences between conditions, no exosuit (NS) and active exosuit (AS) are highlighted by an * in the Average Time Row.

Table S6. Median (50th Percentile) EMG Amplitudes (Mean+SD) reported as a % Maximum Voluntary Isometric Contraction (MVIC) during the Functional Lifting Task for 4 Muscle Groups at 4 epochs (time periods)

Condition	Back Extensors[h,k,a] (% MVIC)	Hip Extensors[k,a] (%MVIC)	Rectus Femoris[a] (%MVIC)	Abdominals (%MVIC)
Time 0-15 ⸸‡✢	N=15	N=15	N=14	N=15
NS	18.1 ± 5.3	11.4 ± 4.0	6.7 ± 4.8	6.1 ± 3.7
AS	15.5 ± 6.0	10.0 ± 4.1	5.4 ± 3.1	6.5 ± 4.3
Time 15-30 ‡✢				
NS	17.8 ± 5.8	10.2 ± 3.7	6.0 ± 3.7	6.0 ± 3.5
AS	14.3 ± 5.7	8.8 ± 3.4	4.8 ± 2.8	6.3 ± 4.2
Time 30-45				
NS	17.6 ± 5.6	9.5 ± 3.3	5.2 ± 3.1	5.1 ± 3.1
AS	14.0 ± 5.1	8.4 ± 3.4	4.1 ± 2.9	5.3 ± 3.3
Time 45-60				
NS	18.0 ± 5.9	9.3 ± 3.3	4.8 ± 3.1	4.5 ± 2.5
AS	13.7 ± 5.2	7.9 ± 3.3	4.0 ± 3.6	4.9 ± 3.2
Average (Time 0-60)				
NS	17.9 ± 5.6*	10.1 ± 3.7*	5.7 ± 3.8*	5.4 ± 3.3
AS	14.4 ± 5.6	8.8 ± 3.6	4.6 ± 3.2	5.8 ± 3.8

Statistics	F-Score	probability value
Condition	F(1,3506)=158.2	**p<0.001**
Epoch	F(3,3506)=39.4	**p<0.001**
MGroup	F(3,3506)=2083.8	**p<0.001**
Cond*Epoch	F(3,3506)=0.4	p=0.763
Cond*MGroup	F(3,3506)=52.0	**p<0.001**
Epoch*MGroup	F(9,3506)=1.6	p=0.125
Cond * Epoch * MGroup	F(9,3506)=0.9	p=0.528

N represents the number of participants with complete EMG data for a specific muscle group. Significant main effects and interactions are highlighted in bold, and trends are indicated by an underline. Significant muscle group by condition post-hocs (*) demonstrate when NS differs from AS within a specified muscle group (column) in the average row. Post hoc between muscle groups are denoted by superscript letters to show when the activity of the indicated site is higher than the back (b), hip (h), or knee (k) extensors and the abdominals (a) in the condition (top) row. Superscript symbols denote significant epoch main effects to show if there is higher activity than epoch 1(†), 2 (⸸), 3 (‡) & 4 (☩) in the condition column. Other post hoc symbols are not included in the table.

Table S7. Peak (90th Percentile) EMG Amplitudes (Mean+SD) reported as a % Maximum Voluntary Isometric Contraction (MVIC) during the Functional Lifting Task for 8 Muscle Sites at 4 epochs (time periods).

Condition	T95[AER] (%MVIC)	L16[AERTGB] (%MVIC)	L33[AERTGBL] (%MVIC)	GM[AER] (%MVIC)	BF[AERTG] (%MVIC)	RF[AE] (%MVIC)	URA (%MVIC)	EO[A] (%MVIC)
Time 0-15⸸‡✢	N=15	N=15	N=13	N=15	N=15	N=14	N=15	N=13
NS	31.0 ± 10.4	35.5 ± 11.8	35.9 ± 13.1	32.8 ± 12.8	34.6 ± 12.9	15.4 ± 8.6	8.5 ± 5.1	12.1 ± 6.9
AS	29.7 ± 12.9	29.1 ± 11.4	32.9 ± 14.6	28.9 ± 14.5	32.1 ± 12.0	12.0 ± 6.5	7.8 ± 4.8	14.9 ± 9.0
Time 15-30‡✢								
NS	29.8 ± 10.2	35.9 ± 12.4	33.8 ± 14.5	28.9 ± 11.0	31.8 ± 12.4	12.8 ± 6.9	8.4 ± 4.4	10.9 ± 6.7
AS	25.9 ± 11.7	27.3 ± 12.2	32.3 ± 14.1	25.5 ± 13.5	29.4 ± 11.3	10.0 ± 5.5	7.5 ± 5.1	13.1 ± 8.1
Time 30-45								
NS	28.6 ± 9.6	36.9 ± 12.2	33.6 ± 14.6	27.5 ± 10.2	30.6 ± 11.4	11.7 ± 6.9	7.4 ± 4.1	9.9 ± 6.5
AS	23.9 ± 10.7	27.4 ± 11.7	31.7 ± 14.6	24.7 ± 12.3	27.9 ± 11.3	9.0 ± 6.3	7.0 ± 4.4	11.0 ± 6.6
Time 45-60								
NS	28.2 ± 9.9	38.4 ± 12.7	34.5 ± 15.1	28.0 ± 10.3	29.8 ± 11.3	11.2 ± 7.0	6.8 ± 3.8	8.8 ± 5.2
AS	21.8 ± 10.5	26.2 ± 11.8	32.3 ± 14.9	24.4 ± 12.4	25.8 ± 11.3	8.8 ± 7.2	6.2 ± 4.1	10.0 ± 5.9
Average								
NS	29.4 ± 10.*	36.7 ± 12.3*	34.4 ± 14.3*	29.3 ± 11.3*	31.7 ± 12.1*	12.8 ± 7.6*	7.8 ± 4.4	10.4 ± 6.4
AS	25.3 ± 11.8	27.5 ± 11.8	32.3 ± 14.5	25.8 ± 13.3	28.8 ± 11.7	9.9 ± 6.5	7.1 ± 4.6	12.2 ± 7.7
Statistics	Main effect or Interaction				F-Score		probability value	

Condition	F(1,6834)=218.0	**p<0.001**
Epoch	F(3,6834)=51.8	**p<0.001**
Muscle	F(7,6834)=1433.1	**p<0.001**
Condition * Epoch	F(3,6834)=2.6	p=0.051
Condition * Muscle	F(7,6834)=31.6	**p<0.001**
Epoch * Muscle	F(21,6834)=3.1	**p=0.005**
Condition * Epoch * Muscle	F(21,6834)=1.1	p=0.399

N represents the number of participants with complete data for a specific muscle site. Significant main effects and interactions are highlighted in bold, and an underline indicates trends. Significant muscle group by condition post-hocs (*) demonstrate when NS differs from AS within a specified muscle site (column) in the average row. Post hoc between muscle sites are denoted by superscript letters to show when the activity of the indicated site is higher than the T95 (T), L16 (L), Gluteus Maximus (G), Biceps Femoris (B), Rectus Femoris (R), Upper Rectus Abdominis (A), and External Obliques (E) in the condition (top) row. Superscript symbols denote significant epoch main effects to show if there is higher activity than epoch 1(†), 2 (⸸), 3 (‡) & 4 (⁜) in the condition column. Other post hoc symbols are not included in the table.

Table S8. Median EMG Amplitudes (Mean+SD) reported as a % Maximum Voluntary Isometric Contraction (MVIC) during the Functional Lifting Task for 8 Muscle Sites at 4 epochs (time periods).

Condition	T95[AREGB] (%MVIC)	L16[AREGBT] (%MVIC)	L33[AREGBT] (%MVIC)	GM[AREB] (%MVIC)	BF[ARE] (%MVIC)	RF (%MVIC)	URA (%MVIC)	EO[AR] (%MVIC)
Time 0-15⸸‡✢	N=15	N=15	N=13	N=15	N=15	N=14	N=15	N=13
NS	16.8 ± 5.8	18.3 ± 6.1	18.9 ± 6.8	11.7 ± 4.6	11.1 ± 4.6	6.7 ± 4.8	5.7 ± 3.4	6.9 ± 5.4
AS	16.2 ± 8.0	14.9 ± 6.6	16.6 ± 7.3	10.8 ± 5.2	9.6 ± 4.4	5.4 ± 3.1	5.2 ± 3.3	8.5 ± 7.0
Time 15-30‡✢								
NS	16.3 ± 5.8	18.8 ± 6.9	17.8 ± 7.2	10.7 ± 4.0	9.9 ± 4.5	6.0 ± 3.7	6.0 ± 3.2	6.4 ± 5.0
AS	14.0 ± 7.2	13.9 ± 7.4	15.8 ± 6.8	9.2 ± 4.1	8.7 ± 4.0	4.8 ± 2.8	5.3 ± 3.7	8.0 ± 6.5
Time 30-45								
NS	15.6 ± 5.4	19.1 ± 6.7	17.4 ± 7.3	10.1 ± 3.8	9.1 ± 3.9	5.2 ± 3.1	5.2 ± 3.0	5.4 ± 4.1
AS	12.7 ± 6.1	14.1 ± 6.6	15.4 ± 6.7	8.9 ± 3.7	8.1 ± 3.9	4.1 ± 2.9	4.8 ± 3.1	6.5 ± 5.2
Time 45-60								
NS	15.5 ± 5.6	20.0 ± 7.4	17.8 ± 7.6	10.1 ± 3.8	8.8 ± 3.8	4.8 ± 3.1	4.6 ± 2.5	4.7 ± 3.4
AS	11.6 ± 5.6	13.8 ± 7.0	15.6 ± 7.1	8.7 ± 3.9	7.3 ± 3.6	4.0 ± 3.6	4.4 ± 3.1	5.6 ± 4.4
Average								
NS	16.1 ± 5.7*	19.0 ± 6.8*	18.0 ± 7.2*	10.6 ± 4.1*	9.7 ± 4.3*	5.7 ± 3.8*	5.4 ± 3.1	5.9 ± 4.6
AS	13.6 ± 6.7	14.2 ± 6.9	15.9 ± 7.0	9.4 ± 4.3	8.4 ± 4.1	4.6 ± 3.2	4.9 ± 3.3	7.2 ± 6.0*

Statistics		F-Score	probability value
	Condition	F(1,6834)=216.3	**p<0.001**
	Epoch	F(3,6834)=48.5	**p<0.001**
	Muscle	F(7,6834)=1132.5	**p<0.001**
	Condition * Epoch	F(3,6834)=2.4	p=0.071
	Condition * Muscle	F(7,6834)=35.1	**p<0.001**
	Epoch * Muscle	F(21,6834)=2.9	**p<0.001**
	Condition * Epoch * Muscle	F(21,6834)=1.1	p=0.315

N represents the number of participants with complete data for a specific muscle site. Significant main effects and interactions are highlighted in bold, and an underline indicates trends. Significant muscle group by condition post-hocs (*) demonstrate when NS differs from AS within a specified muscle site (column) in the average row. Post hoc between muscle sites are denoted by superscript letters to show when the activity of the indicated site is higher than the T95 (T), L16 (L), Gluteus Maximus (G), Biceps Femoris (B), Rectus Femoris (R), Upper Rectus Abdominis (A), and External Obliques (E) in the condition (top) row. Superscript symbols denote significant epoch main effects to show if there is higher activity than epoch 1(†), 2 (⸸), 3 (‡) & 4 (✢) in the condition column. Other post hoc symbols are not included in the table.

Table S9. Peak (90th Percentile) EMG Amplitudes (Mean+SD) reported as a % Maximum Voluntary Isometric Contraction (MVIC) during the Functional Lifting Task for Lift Type/ Box Position.

Mass Position	Close High		Close Low		Far High		Far Low	
Lift Type	Lift	Lower	Lift	Lower	Lift	Lower	Lift	Lower
Back Extensor	N=15	N=15	N=15	N=15	N=15	N=15	N=15	N=15
NS	35.5 ± 11.2	28.7 ± 8.5	38.4 ± 11.7	30.8 ± 8.2	38.9 ± 11.4	30.7 ± 9.2	38.7 ± 11.7	30.6 ± 8.2
AS	29.5 ± 10.0	23.9 ± 8.6	30.8 ± 10.2	25.8 ± 9.2	31.8 ± 10.9	25.5 ± 9.3	31.2 ± 10.8	25.4 ± 9.0
Hip Extensor	N=15	N=15	N=15	N=15	N=15	N=15	N=15	N=15
NS	32.3 ± 11.3	24.3 ± 6.6	36.3 ± 11.1	29.3 ± 8.6	35.3 ± 10.6	22.8 ± 6.3	37.3 ± 11.8	26.5 ± 7.3
AS	29.0 ± 10.3	21.4 ± 7.5	33.0 ± 10.3	26.6 ± 8.8	32.2 ± 10.9	20.5 ± 6.3	33.6 ± 11.4	23.1 ± 7.2
Rectus Femoris	N=14	N=14	N=14	N=14	N=14	N=14	N=14	N=14
NS	13.6 ± 7.2	10.3 ± 6.0	15.2 ± 7.2	12.1 ± 7.4	13.1 ± 7.8	10.6 ± 6.6	14.5 ± 8.2	12.2 ± 7.3
AS	10.1 ± 5.9	7.7 ± 5.3	11.5 ± 6.6	8.7 ± 5.8	12.0 ± 7.1	7.6 ± 5.0	13.1 ± 8.4	8.8 ± 5.3
Abdominals	N=15	N=15	N=15	N=15	N=15	N=15	N=15	N=15
NS	9.3 ± 4.7	8.3 ± 4.8	8.8 ± 4.6	8.3 ± 4.9	9.2 ± 4.8	8.0 ± 4.3	9.3 ± 4.7	8.3 ± 4.3
AS	9.5 ± 5.0	8.6 ± 4.8	9.2 ± 4.9	8.6 ± 4.8	10.3 ± 5.5	8.4 ± 4.8	10.0 ± 5.2	8.8 ± 4.8

Table S10. Median EMG Amplitudes (Mean+SD) reported as a % Maximum Voluntary Isometric Contraction (MVIC) during the Functional Lifting Task for Lift Type/ Box Position.

Mass Position	Close High		Close Low		Far High		Far Low	
Lift Type	Lift	Lower	Lift	Lower	Lift	Lower	Lift	Lower
Back Extensor	N=15	N=15	N=15	N=15	N=15	N=15	N=15	N=15
NS	19.3 ± 11.2	17.3 ± 5.7	18.6 ± 5.6	18.7 ± 5.4	17.7 ± 5.2	17.9 ± 5.8	16.2 ± 4.9	17.6 ± 5.2
AS	15.6 ± 5.6	13.9 ± 5.2	14.5 ± 5.3	14.7 ± 5.3	15.1 ± 6.6	14.2 ± 5.4	13.1 ± 5.8	13.9 ± 5.1
Hip Extensor	N=15	N=15	N=15	N=15	N=15	N=15	N=15	N=15
NS	9.6 ± 3.9	10.4 ± 3.3	9.7 ± 3.8	11.1 ± 4.3	10.6 ± 4.0	9.6 ± 3.1	9.9 ± 3.6	9.8 ± 3.5
AS	8.4 ± 3.7	9.1 ± 3.6	8.4 ± 3.6	9.9 ± 4.2	9.3 ± 3.6	8.3 ± 3.3	8.5 ± 3.4	8.5 ± 3.1
Rectus Femoris	N=14	N=14	N=14	N=14	N=14	N=14	N=14	N=14
NS	5.8 ± 3.5	4.5 ± 3.3	6.4 ± 4.1	5.5 ± 4.1	6.2 ± 3.6	4.8 ± 3.2	6.5 ± 4.1	5.7 ± 3.8
AS	4.6 ± 2.7	3.4 ± 2.5	5.1 ± 3.2	4.2 ± 3.1	5.5 ± 3.2	3.8 ± 3.1	5.7 ± 3.9	4.3 ± 2.8
Abdominals	N=15	N=15	N=15	N=15	N=15	N=15	N=15	N=15
NS	5.5 ± 3.2	5.5 ± 3.5	5.4 ± 3.3	5.3 ± 3.4	5.5 ± 3.4	5.3 ± 3.2	5.5 ± 3.3	5.3 ± 3.2
AS	5.8 ± 3.8	5.7 ± 3.9	5.7 ± 3.8	5.6 ± 3.9	6.0 ± 3.9	5.6 ± 3.8	5.9 ± 4.0	5.6 ± 3.7

Table S11. Demographics of the 15 participants included in the order-picking task experiment data represent mean ± standard deviation.

Participants	Age (Years)	Mass (kg)	Height (m)	BMI (kg m-2)
N=15 (Female: 4, 27%)	31 ± 4	73 ± 12	172 ± 13	25 ± 5

Table S12. Demographics of the 10 participants included in the system usability experiment data represent mean ± standard deviation.

Participants	Age (Years)	Mass (kg)	Height (cm)	BMI (kg m-2)
N=10 (Female: 1, 10%)	26 ± 5	76 ± 10	181 ± 11	23 ± 3

5.0 Supplemental Movies

Movie S1.
Usability test.

Movie S2.
Order picking test.
