# Supplementary material for: Lightweight active back exosuit reduces muscular effort during an hour-long order picking task
Source: Commun Eng. 2024 Feb 23;3:35. doi: 10.1038/s44172-024-00180-w (PMC10955849; doi:10.1038/s44172-024-00180-w)
Supplement: Supplementary file 2 — Description of Additional Supplementary Files [file 44172_2024_180_MOESM2_ESM.pdf]

Description of Additional Supplementary Files

File name: Supplementary Movie 1

Description: System Usability Assessment Task

File name: Supplementary Movie 2

Description: Order Picking Task Demonstration
