# Supplementary material for: Lightweight active back exosuit reduces muscular effort during an hour-long order picking task
Source: Commun Eng. 2024 Feb 23;3:35. doi: 10.1038/s44172-024-00180-w (PMC10955849; doi:10.1038/s44172-024-00180-w)
Supplement: Supplementary file 5 — Reporting Summary [file 44172_2024_180_MOESM5_ESM.pdf]

nature portfolio

Corresponding author(s): Conor J. Walsh

Last updated by author(s): Jan 2, 2024

Reporting Summary

Nature Portfolio wishes to improve the reproducibility of the work that we publish. This form provides structure for consistency and transparency in reporting. For further information on Nature Portfolio policies, see our Editorial Policies and the Editorial Policy Checklist.

Statistics

For all statistical analyses, confirm that the following items are present in the figure legend, table legend, main text, or Methods section.

n/a	Confirmed	
☐	☒	The exact sample size (*n*) for each experimental group/condition, given as a discrete number and unit of measurement
☐	☒	A statement on whether measurements were taken from distinct samples or whether the same sample was measured repeatedly
☐	☒	The statistical test(s) used AND whether they are one- or two-sided<br>*Only common tests should be described solely by name; describe more complex techniques in the Methods section.*
☒	☐	A description of all covariates tested
☐	☒	A description of any assumptions or corrections, such as tests of normality and adjustment for multiple comparisons
☐	☒	A full description of the statistical parameters including central tendency (e.g. means) or other basic estimates (e.g. regression coefficient) AND variation (e.g. standard deviation) or associated estimates of uncertainty (e.g. confidence intervals)
☐	☒	For null hypothesis testing, the test statistic (e.g. F, t, r) with confidence intervals, effect sizes, degrees of freedom and P value noted<br>*Give P values as exact values whenever suitable.*
☒	☐	For Bayesian analysis, information on the choice of priors and Markov chain Monte Carlo settings
☒	☐	For hierarchical and complex designs, identification of the appropriate level for tests and full reporting of outcomes
☐	☒	Estimates of effect sizes (e.g. Cohen's *d*, Pearson's *r*), indicating how they were calculated

Our web collection on statistics for biologists contains articles on many of the points above.

Software and code

Policy information about availability of computer code

Data collection	Qualisys Track Manager (Version 2020.2, QualisysTM, Goteborg, Sweden); EMGWorks Software (Delsys Inc., Natick, MA)
Data analysis	Matlab code (Version 2020b, The Math WorksTM, Natick, MA, USA) for filtering, event normalizing, averaging and calculating outcome measures for time-varying inertial measurement unit, load cell, and electromyography data. Minitab 19 (Minitab LLC, State College, PA) were used for statistical analysis.

For manuscripts utilizing custom algorithms or software that are central to the research but not yet described in published literature, software must be made available to editors and reviewers. We strongly encourage code deposition in a community repository (e.g. GitHub). See the Nature Portfolio guidelines for submitting code & software for further information.

Data

Policy information about availability of data

All manuscripts must include a data availability statement. This statement should provide the following information, where applicable:

- Accession codes, unique identifiers, or web links for publicly available datasets
- A description of any restrictions on data availability
- For clinical datasets or third party data, please ensure that the statement adheres to our policy

The derived data that support the findings of this study are available from the corresponding author (C.J.W) upon reasonable request.

Human research participants

Policy information about studies involving human research participants and Sex and Gender in Research.

Reporting on sex and gender	Participant sex was self-reported as mentioned in the supplementary methods. The primary study included 11 men and 4 women. Given the small number of women sex was not analyzed separately. Data were pooled across men and women to improve generalization of the data.
Population characteristics	Age, sex, mass, and weight were collected from participants. The studies repeated measure design balanced characteristics confounders, hence they were not included as co-variates at the risk of over fitting the data.
Recruitment	Participants were recruited via internal advertisements and word-of-mouth recruitment on a voluntary basis from a university campus. Data is subjected to self-selection bias of those interested in wearing the study device (back exosuit).
Ethics oversight	The study was conducted under a protocol approved by Harvard Medical School's Institutional Review Board (IRB18-0960).

Note that full information on the approval of the study protocol must also be provided in the manuscript.

Field-specific reporting

Please select the one below that is the best fit for your research. If you are not sure, read the appropriate sections before making your selection.

- [x] Life sciences
- [ ] Behavioural & social sciences
- [ ] Ecological, evolutionary & environmental sciences

For a reference copy of the document with all sections, see nature.com/documents/nr-reporting-summary-flat.pdf

Life sciences study design

All studies must disclose on these points even when the disclosure is negative.

Sample size	To ensure study power, sample size was calculated to determine exosuit differences in peak back extensor EMG amplitudes. Studies cited in the primary study demonstrate large EMG differences (effect size: Cohen's d=1.08) between exosuit and no-exosuit conditions. Fourteen participants would be required detect condition differences with 80% power and an α=0.05.
Data exclusions	Data was retained from all participants. Within a participant, EMG data could be excluded if the sensor fell off or was exposed to excessive motion artifact. The number of EMG sensors with complete data are included all main manuscript and supplementary tables.
Replication	Overall reductions in peak and median EMG are consistent with other studies both within our laboratory and across laboratories. Study findings are interpreted to these data in the study discussion.
Randomization	The study had a repeated measure design. However, the condition in which participants performed the order picking task was randomized using a counter-balanced Latin square design. An equal number of participants started the task with or without the exosuit on their first data collection session.
Blinding	The back exosuit is an external device that provides noticeable assistance. The device had to be donned and worn in the exosuit condition. Participants and investigators were not blind to the participants exosuit status.

Reporting for specific materials, systems and methods

We require information from authors about some types of materials, experimental systems and methods used in many studies. Here, indicate whether each material, system or method listed is relevant to your study. If you are not sure if a list item applies to your research, read the appropriate section before selecting a response.

Materials & experimental systems

n/a	Involved in the study	
☒	☐	Antibodies
☒	☐	Eukaryotic cell lines
☒	☐	Palaeontology and archaeology
☒	☐	Animals and other organisms
☒	☐	Clinical data
☒	☐	Dual use research of concern

Methods

n/a	Involved in the study	
☒	☐	ChIP-seq
☒	☐	Flow cytometry
☒	☐	MRI-based neuroimaging
